# Association of Hemostatic Markers with Atrial Fibrillation: A Meta-Analysis and Meta-Regression

**DOI:** 10.1371/journal.pone.0124716

**Published:** 2015-04-17

**Authors:** Na Wu, Shifei Tong, Ying Xiang, Long Wu, Bin Xu, Yao Zhang, Xiangyu Ma, Yafei Li, Zhiyuan Song, Li Zhong

**Affiliations:** 1 Department of Epidemiology, College of Preventive Medicine, Third Military Medical University, Chongqing, People’s Republic of China; 2 Evidence-based Medicine and Clinical Epidemiology Center, Third Military Medical University, Chongqing, People’s Republic of China; 3 Department of Cardiology, Southwest Hospital, Third Military Medical University, Chongqing, People’s Republic of China; Harvard Medical School, UNITED STATES

## Abstract

**Background:**

There is growing evidence that indicates the presence of a prothrombotic state in atrial fibrillation (AF). However, the role of hemostatic markers in AF remains inconclusive.

**Methods:**

We conducted a meta-analysis of observational studies to evaluate the association between hemostatic markers and AF. A meta-regression was performed to explore potential sources of heterogeneity.

**Results:**

A total of 59 studies met our inclusion criteria for the meta-analysis. For platelet activation, increased circulating platelet factor-4, β-thromboglobulin (BTG) and P-selectin were significantly higher in AF cases compared with controls (standardized mean difference [SMD][95% confidence interval (CI)]: 1.72[0.96–2.49], 1.61[1.03–2.19] and 0.50[0.23–0.77], respectively). For coagulation activation, increased levels of plasma D-dimer, fibrinogen, thrombin-antithrombin, prothrombin fragment 1+2, and antithrombin-III were significantly associated with AF (SMD[95% CI]: 1.82[1.38–2.26], 0.72[0.55–0.89], 0.42[0.13–0.72], 1.00 [0.00–1.99] and 1.38[0.16–2.60], respectively). For fibrinolytic function, tissue-type plasminogen activator and plasminogen activator inhibitor-1 were significantly increased in AF cases compared with controls (SMD[95% CI]: 0.86[0.04–1.67] and 0.87[0.28–1.47], respectively) but the associations became nonsignificant after performing subgroup analysis by anticoagulants treatment status. For endothelial function, increased von Willebrand factor was significantly associated with AF (SMD, 0.79; 95% CI, 0.60–0.99); however, no association was observed for soluble thrombomodulin (SMD, 0.60; 95% CI, -0.13–1.33).

**Conclusions:**

Increased circulating hemostatic factors (PF-4, BTG, P-selectin, D-dimer, fibrinogen, TAT, F1+2, AT- III, and vWf) are significantly associated with AF. Future research is necessary to elucidate the precise mechanism of the prothrombotic state and how hemostatic markers promote thromboembolism in AF.

## Introduction

The prevalence of atrial fibrillation (AF) is estimated to be 0.4% to 1.0% in the general population and is increasing [1]. It confers a high risk of mortality and morbidity from strokes and thromboembolisms [2]. The pathophysiological mechanism that leads to strokes and thromboembolisms in AF is multifactorial. Stasis in the poorly contractile left atrium is partially responsible, but there is increasing evidence that indicates the presence of a prothrombotic or hypercoagulable state in AF [3]. Over 150 years ago, Rudolf Virchow proposed a triad of events that are necessary for thrombus formation, including blood flow abnormalities, blood vessel wall abnormalities and blood constituents [4]. In the 21st century, Virchow’s triad has been revised as follows: endothelial or endocardial damage or dysfunction (and related structural abnormal changes); abnormal blood stasis; and abnormal hemostasis, platelets, and fibrinolysis [2, 3].

An increasing number of studies have been performed to delineate hemostatic function in AF. The abnormalities observed in AF primarily include abnormal endothelial function, platelet and coagulation activation, and abnormal fibrinolysis. For coagulation and platelet activation, some studies have demonstrated that coagulation and platelet activation markers were elevated in AF [5–7]. However, other studies have failed to demonstrate these associations [8, 9]. The fibrinolytic system is usually activated when a thrombus is formed, but it is still controversial whether a hyperfibrinolytic or hypofibrinolytic state is related to AF [10–12]. It is also unclear whether endothelial dysfunction, indicated by circulating markers of endothelial origin, is associated with AF [13, 14].

Identifying the specific hemostatic markers that are associated with AF is important for understanding AF etiology, development and prognosis. Therefore, we conducted a meta-analysis to evaluate the associations between hemostatic markers (including markers of platelet activation, coagulation activation, fibrinolytic function, and endothelial function) and AF.

## Methods

### Search Strategy

This meta-analysis was performed according to the guidelines presented in the Preferred Reporting Items for Systematic Reviews and Meta-Analyses (PRISMA) [15]. We used electronic database and manual searches to identify relevant studies. The PubMed, EMBASE, Web of Science, and Chinese Biomedical Literature (CBM) databases were systematically searched for studies published up to July 2013 using a web-based search engine. The search terms used to identify relevant studies were “platelet”, “platelet factor-4”, “β-thromboglobulin”, “P-selectin”, “D-dimer”, “fibrinogen”, “prothrombin fragment 1+2”, “thrombin-antithrombin”, “antithrombin-III”, “a_2_-antiplasmin”, “fibrinopeptide A”, “tissue-type plasminogen activator”, “urokinase-type plasminogen activator”, “plasminogen activator inhibitor”, “plasmin-antiplasmin complex”, “von Willebrand factor” or “soluble thrombomodulin”, and “atrial fibrillation”. We also manually searched journals and the reference lists of all retrieved articles. Additionally, relevant review articles were also cross-referenced. The literature retrieval was performed in duplication by two independent reviewers (N.W. and S. T.).

### Study selection

Human studies, regardless of sample size, were included if they met the following criteria: (1) the study design was a case-control study (retrospective or nested case-control) or cohort study (retrospective or prospective cohort study) and (2) the study investigated the association between hemostatic markers (including markers of endothelial function, platelet activation, coagulation activation, and fibrinolytic function) and AF.

When multiple publications were based on the same or overlapping data, we used the most recent or largest population as recommended by Little et al [16]. The number of participants in groups, mean hemostatic marker levels and standard deviations (SD) had to be provided or could be converted from the median and range data or determined from figures. For studies that did not have adequate data, we contacted the authors to obtain the unpublished results; if the author was not able to provide the necessary data, these studies were excluded. Certain hemostatic markers, such as urokinase-type plasminogen activator, a_2_-antiplasmin, plasmin-antiplasmin complex and fibrinopeptide A, were investigated in less than two studies and were also excluded from this meta-analysis.

### Quality Assessment

Two reviewers (N.W. and S. T.) independently assessed the study quality using the primary criteria for nonrandomized studies described in the Newcastle-Ottawa scale [17]. A “score system” was developed based on the Newcastle-Ottawa criteria (S1 Table). The total scores ranged from 0 (worst) to 9 (best) for case-control or cohort studies. A score consensus was reached after discussion to resolve any disagreements.

### Data Extraction

Using standard data extraction forms, two reviewers (N.W. and Y.X.) extracted data from the relevant studies. We extracted publication information (first author’s name, publication year, country, study design), participant characteristics (mean age of participants, gender, sample size, type of study e.g., case/control, anticoagulants treatment status, coexistent cardiovascular disease and risk factors), the type of AF and hemostatic marker levels (the mean and SD of each group). Any disagreements were resolved by consensus with a third reviewer (L.Z.).

If the study provided the medians and ranges instead of the means and SDs, we imputed the means and SDs using the method developed by Hozo et al [18]. For studies that reported interquartile ranges instead of the range, we multiplied the difference between the median, upper and lower ends of the interquartile range by 2 and added or subtracted the product from the median to estimate the lower and upper values of the range as described by Liu et al [19]. For studies that provided only figures and when the exact data could not be obtained after contacting the authors, we used the program Engauge Digitizer 4.1 (M. Mitchell, Engauge Digitizer, http://digitizer.sourceforge.net) to read the exact means and SDs from the figures. This program was able to read the exact values by digitizing the data points in an image file after manually setting the axis coordinates.

### Statistical analysis

The standardized mean difference (SMD) was used as a summary statistic in the meta-analysis. SMD is the difference in mean outcomes between groups divided by the standard deviation of the outcome among participants. The SMD method is commonly used when a variety of methods and units are used to measure hemostatic marker levels among different studies. In this circumstance, it was necessary to standardize the results of the studies to a uniform scale before they were able to be combined. The statistical heterogeneity across studies was assessed using the *I* ^2^ statistic, which describes the proportion of total variation across studies that is due to heterogeneity rather than chance [20]. An *I* ^2^≥50% suggests that there is significant heterogeneity between studies. The SMD was pooled using a random effects model to manage heterogeneity when the heterogeneity was significant (*I* ^2^ ≥50%); otherwise, a fixed effects model was applied. We evaluated potential publication bias using funnel plots and Begg’s tests [21].

A weighted random-effect meta-regression analysis was performed to examine potential sources of heterogeneity and potential confounding factors. The weight for each trial was equal to the inverse of the sum of the within trial variance and the residual between trial variance. The restricted maximum likelihood (REML) method was used to estimate the residual between trial variance. The true effect of each included study was treated as random-effect, and the true effects of included studies are a random sample of the relevant distribution of true effects [22]. Based on prior research [7, 23–25], we examined specific between-study characteristics which might influence the level of hemostatic markers and attribute to heterogeneity, including study design (case-control study vs. cohort study), publication year (before 2000 vs. after 2000), mean age (under 60 years vs. at least 60 years), type of AF (paroxysmal AF which is defined as arrhythmia terminates spontaneously vs. persistent AF which refers to AF sustained beyond 7 days and permanent AF denoting long-standing AF which lasts more than 1 year [1]), cardiovascular disease and risk factors, including hypertension, coronary artery disease, cerebrovascular accidents, diabetes mellitus, smoking and gender (the ratio of the proportion of subjects who have the investigated factors between AF patients and controls). We performed univariate meta-regressions and these factors were entered into the meta-regression model separately. A multivariate meta-regression was also conducted when more than one characteristic was significantly (*P*<0.05) in univariate model. A subgroup analysis was performed by categorical variables which were significant in the univariate meta-regression model to explore how these factors influenced the level of hemostatic markers. Considering anticoagulant treatment had great impact on levels of hemostatic markers, we also carried out a subgroup analysis by anticoagulants treatment status as follows: Subgroup 1, there was significant difference in the proportion of subjects received anticoagulants between AF patients and controls; Subgroup 2, no patient received anticoagulants in both groups; Subgroup 3, all participants were anticoagulated in both groups; Subgroup 4, there was no significant difference in the proportion of anticoagulation treatment in both groups (excluding the Subgroup 2 and 3); Subgroup 5, anticoagulation information was not available in both groups. The significance level for all analyses was set at *P*<0.05. Statistical analyses were performed using the STATA software package (version 11.0, College Station, TX).

## Results

### Description of studies

**Fig 1:**
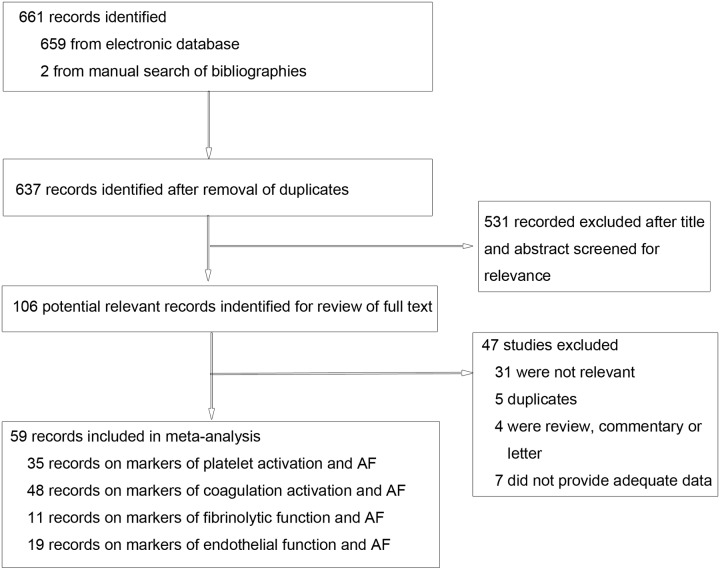
Flow diagram of the literature search and study selection.

A total of 661 records were retrieved after the primary literature search. After screening the titles and abstracts, 531 studies were excluded; 106 potentially relevant full-text articles were reviewed, and 59 studies were included in the meta-analysis (Fig 1). Of the 59 included studies, 35 studies investigated the association between platelet activation markers and AF, with a total of 2730 cases and 2371 controls [5–9, 13, 23, 24, 26–52]. There were 48 studies examining the association between coagulation activation markers and AF, including 5412 AF cases and 29292 controls [5, 7, 8, 11–14, 24, 25, 27, 29, 31, 33, 34, 36–39, 41–43, 45–48, 50–72]. A total of 11 studies investigated the association between fibrinolytic function markers and AF, with 631 patients and 6558 controls [11–13, 31, 45, 50, 59, 61, 63, 67, 72]. Finally, 19 studies examined the association between endothelial function markers and AF, with 2502 cases and 15289 controls [7, 13, 14, 24, 27, 31, 34, 37, 41–43, 46, 50–52, 57, 59, 64, 71]. The mean age of AF patients ranged from 37.0 to 81.5. All case-control and cohort studies had a quality score of at least 6 (S2 Table).

### Platelet activation

**Fig 2:**
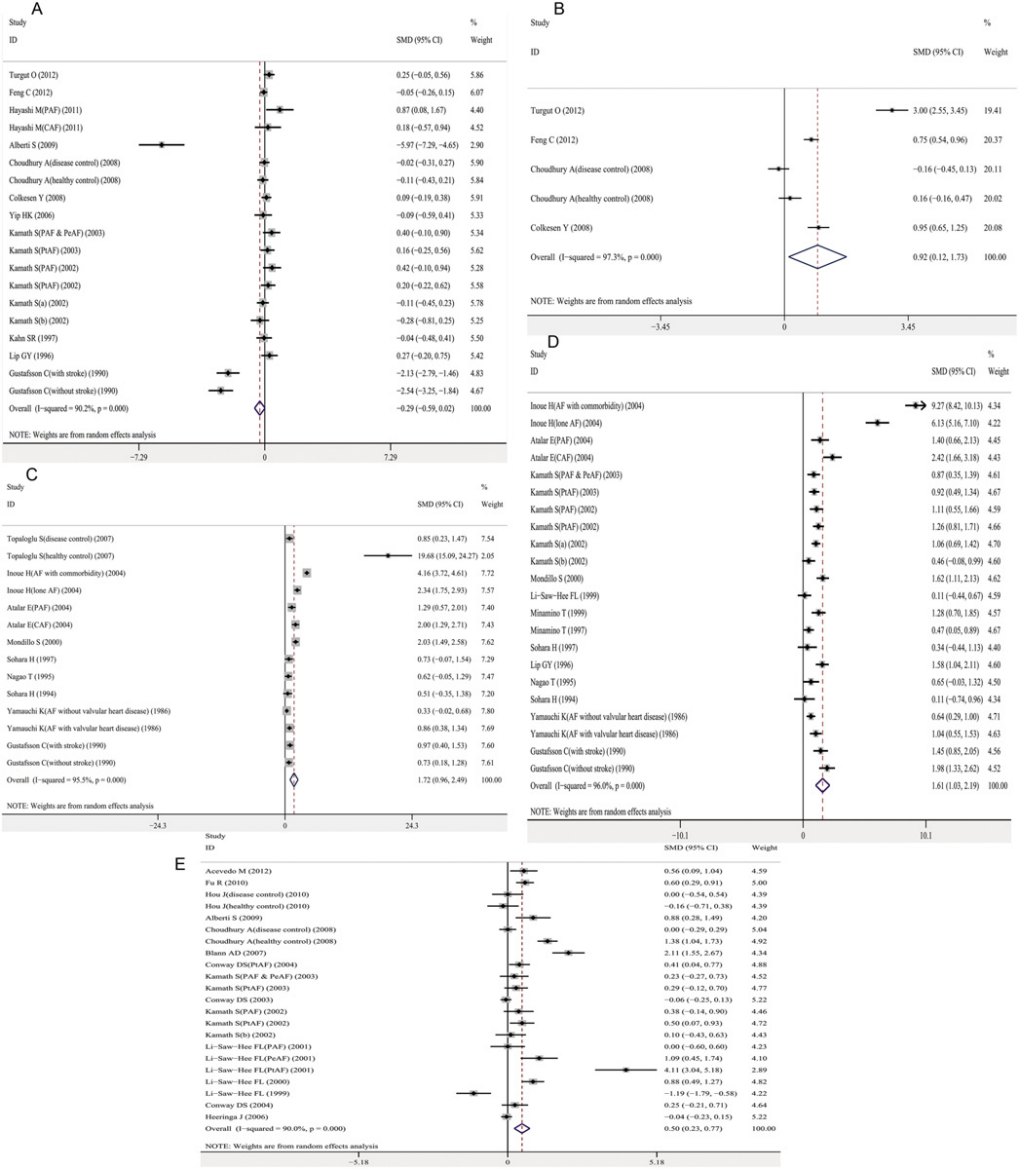
Association between platelet activation markers and AF. A. platelet count and AF; B. Mean platelet volume and AF; C. Platelet factor-4 and AF; D. β-thromboglobulin and AF; E. P-selectin and AF. Forest plots of SMD and overall SMD with 95% CI between AF cases and controls. Black diamonds indicate the SMD, with the size of the square inversely proportional to its variance, and horizontal lines represent the 95% CI. The pooled results are indicated by the black hollow diamond. AF, atrial fibrillation; MPV, mean platelet volume; PF-4, platelet factor-4; BTG, β-thromboglobulin; PAF, paroxysmal AF; PeAF, persistent AF; PtAF, permanent AF; CAF, chronic AF; SMD, standardized mean difference.

The meta-analysis of 14 studies determined that platelet count was not significantly different between AF cases and controls with a pooled SMD -0.29 (95% CI, -0.59–0.02; *P* = 0.066) (Fig 2A). Mean platelet volume (MPV), circulating levels of platelet factor-4 (PF-4), β-thromboglobulin (BTG) and P-selectin were higher in AF cases than controls, with a pooled SMD of 0.92 (95% CI, 0.12–1.73; *P* = 0.025), 1.72 (95% CI, 0.96–2.49; *P*<0.001), 1.61 (95% CI, 1.03–2.19; *P*<0.001), and 0.50 (95% CI, 0.23–0.77; *P*<0.001), respectively (Fig 2B–2E).

There was significant heterogeneity for studies investigating platelet activation markers, with an I^2^ above 90% (Table 1). In the univariate meta-regression analyses, three factors were associated with heterogeneity for platelet count: type of AF, hypertension, and coronary artery disease. The multivariate meta-regression could not be performed for platelet count because the limited number of included studies. We did not find an association between all the potential factors and heterogeneity for MPV, PF-4, BTG and P-selectin (S3 Table). A subgroup analysis indicated that there was no significant association between platelet count and paroxysmal or permanent AF (S1 Fig). The heterogeneity was significantly reduced among the three paroxysmal AF studies with an I^2^ = 49.3% (*P* = 0.139) and two permanent AF studies with an I^2^ = 0.0% (*P* = 0.895) (S1 Fig). The results of univariate meta-analysis were not materially altered for platelet count, PF-4, BTG and P-selectin after grouping by anticoagulants treatment status (data not shown). However, the association between MPV and AF vanished in studies with and without different proportion of people anticoagulated in two compared groups (S2 Fig).

**Table 1:** Summary results of meta-analysis by hemostatic markers.

Hemostatic marker	Pooled SMD with 95% CI	Test for overall effect (P value)	Heterogeneity	
			Q-test (P value)	I^2^ (%)
Platelet activation				
Platelet count	-0.29 (-0.59–0.02)	0.066	<0.001	90.2
MPV	0.92 (0.12–1.73)	0.025	<0.001	97.3
PF-4	1.72 (0.96–2.49)	<0.001	<0.001	95.5
BTG	1.61 (1.03–2.19)	<0.001	<0.001	96.0
P-selectin	0.50 (0.23–0.77)	<0.001	<0.001	90.0
Coagulation activation				
D-dimer	1.82 (1.38–2.26)	<0.001	<0.001	96.6
Fibrinogen	0.72 (0.55–0.89)	<0.001	<0.001	94.0
TAT	0.42 (0.13–0.72)	0.005	0.298	18.3
F1+2	1.00 (0.00–1.99)	0.049	<0.001	97.7
AT- III	1.38 (0.16–2.60)	0.027	<0.001	95.6
Fibrinolytic function				
tPA	0.86 (0.04, 1.67)	0.041	<0.001	96.7
PAI-1	0.87 (0.28–1.47)	0.004	<0.001	96.3
Endothelial function				
vWf	0.79 (0.60–0.99)	<0.001	<0.001	90.1
sTM	0.60 (-0.13–1.33)	0.107	<0.001	91.6

AF, atrial fibrillation; MPV, mean platelet volume; PF-4, platelet factor 4; BTG, β-thromboglobulin; P-sel, P-selectin; Fib, fibrinogen; TAT, thrombin—antithrombin; F1+2, prothombin fragments 1+2; AT- III, Antithrombin III; tPA, tissue plasminogen activator; PAI-1, plasminogen activator inhibitor-1; vWf, vonWillebrand factor; sTM, soluble thrombomodulin; SMD, standardized mean difference; CI, confidence interval.

### Coagulation activation

**Fig 3:**
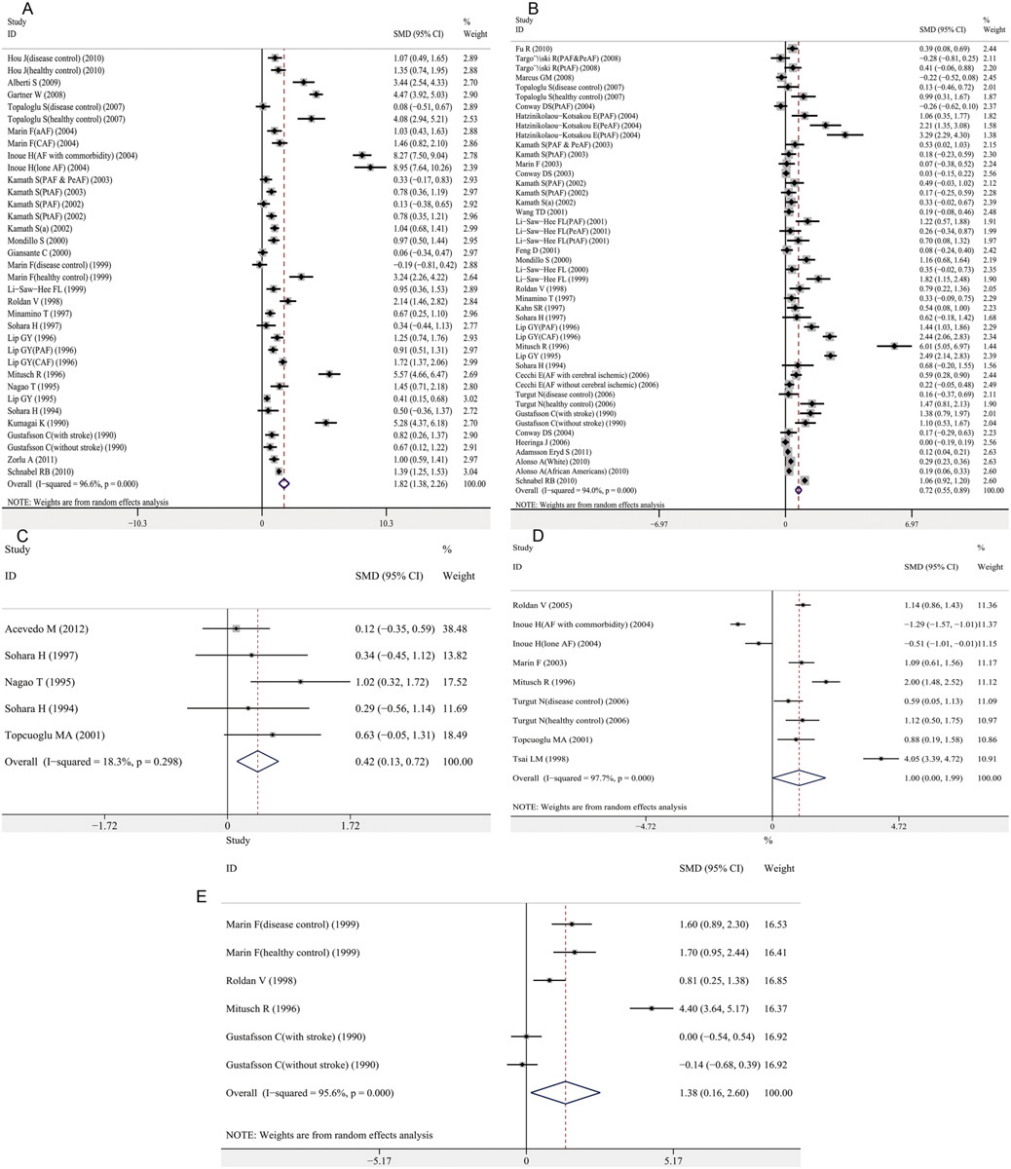
Association between coagulation activation markers and AF. A. D-dimer and AF; B. fibrinogen and AF; C. Thrombin-antithrombin and AF; D. Prothrombin fragment 1+2 and AF; E. Antithrombin- III and AF. Forest plots of SMD and overall SMD with 95% CI between AF cases and controls. Black diamonds indicate the SMD, with the size of the square inversely proportional to its variance, and horizontal lines represent the 95% CI. The pooled results are indicated by the black hollow diamond. AF, atrial fibrillation; TAT, thrombin-antithrombin; F1+2, prothrombin fragment 1+2; AT- III, antithrombin- III; PAF, paroxysmal AF; PeAF, persistent AF; PtAF, permanent AF; CAF, chronic AF; aAF, acute AF; SMD, standardized mean difference.

Coagulation activation markers, including plasma D-dimer, fibrinogen, thrombin-antithrombin (TAT), prothrombin fragment 1+2 (F1+2), and antithrombin- III (AT- III), were significantly higher in patients with AF than controls, with a pooled SMD of 1.82 (95% CI, 1.38–2.26; *P*<0.001), 0.72 (95% CI, 0.55–0.89; *P*<0.001), 0.42 (95% CI, 0.13–0.72; *P* = 0.005), 1.00 (95% CI, 0.00–1.99; *P* = 0.049), and 1.38 (95% CI, 0.16–2.60; *P* = 0.027), respectively (Fig 3A–3E). There was significant heterogeneity between the studies investigating D-dimer, fibrinogen, F1+2, and AT- III (Table 1). According to the univariate meta-regression results, the type of AF was associated with heterogeneity for D-dimer. The publication year and gender were associated with heterogeneity for fibrinogen (S3 Table), and multivariate meta-analysis showed similar result with univariate model, indicating publication year and gender were significantly associated with heterogeneity for fibrinogen. The proportion of between-study variance explained by these two covariates was 41.26% (S4 Table). A subgroup analysis demonstrated that the association between D-dimer and AF was significant in paroxysmal, persistent and permanent AF (S3 Fig). After grouping by publication year, the associations between fibrinogen and AF were significant for studies published after 2000 and those published before 2000 (S4 Fig). Results of subgroup analyses by anticoagulants treatment status for D-dimer, fibrinogen, TAT, F1+2 and AT- III were consistent with the overall effects (data not shown). A meta-regression analysis was not performed for TAT, F1+2, and AT- III because of a limited number of studies.

### Fibrinolytic function

**Fig 4:**
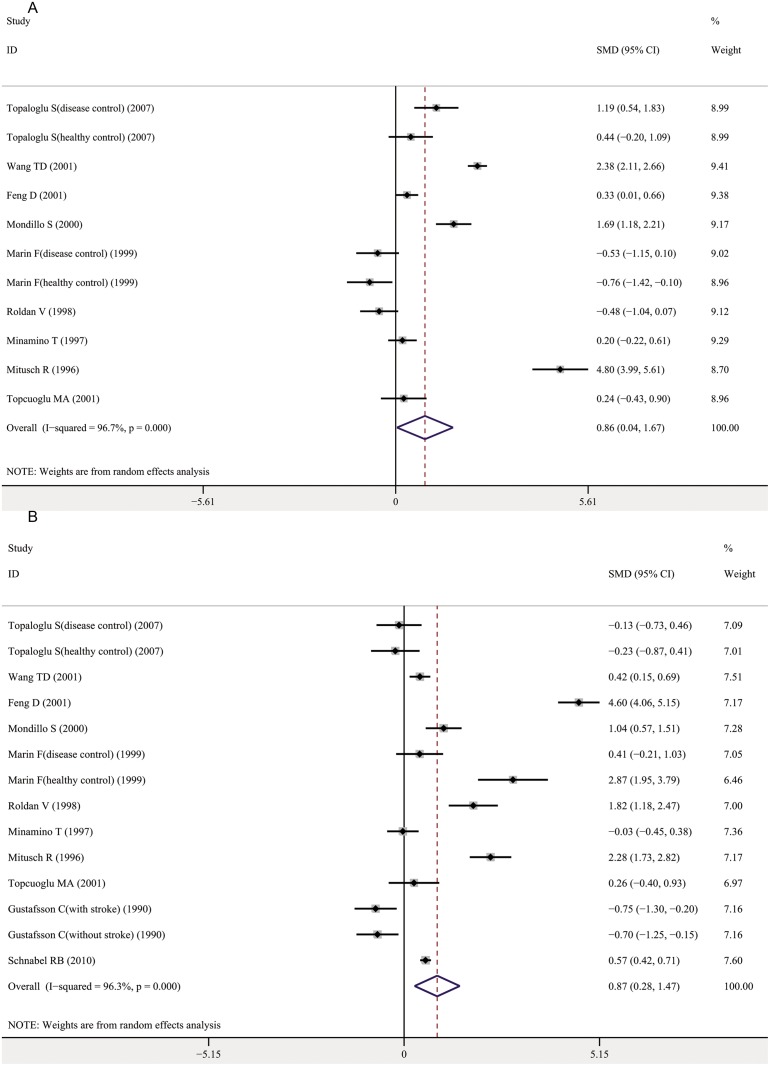
Association between fibrinolytic function makers and AF. A. Tissue-type plasminogen activator and AF; B. Plasminogen activator inhibitor-1 and AF. Forest plots of SMD and overall SMD with 95% CI between AF cases and controls. Black diamonds indicate the SMD, with the size of the square inversely proportional to its variance, and horizontal lines represent the 95% CI. The pooled results are indicated by the black hollow diamond. AF, atrial fibrillation; tPA, tissue-type plasminogen activator; PAI-1, plasminogen activator inhibitor-1; SMD, standardized mean difference.

The indicators of fibrinolytic function included in this meta-analysis were tissue-type plasminogen activator (tPA) and plasminogen activator inhibitor-1 (PAI-1). A meta-analysis of 9 studies for tPA and 11 studies for PAI-1 determined that both markers were significantly increased in AF cases compared with controls, with a pooled SMD of 0.86 (95% CI, 0.04–1.67; *P* = 0.041) and 0.87 (95% CI, 0.28–1.47; *P* = 0.004), respectively (Fig 4A and 4B). A subgroup analysis stratified by anticoagulants treatment status was performed. For both tPA and PAI-1, the associations became nonsignificant for all subgroups (S5 Fig, S6 Fig). There was significant heterogeneity among the studies investigating tPA and PAI-1, with an I^2^ of approximately 96% (Table 1). However, univariate meta-regression analyses did not identify any potential sources of heterogeneity for tPA and PAI-1(S3 Table).

### Endothelial function

**Fig 5:**
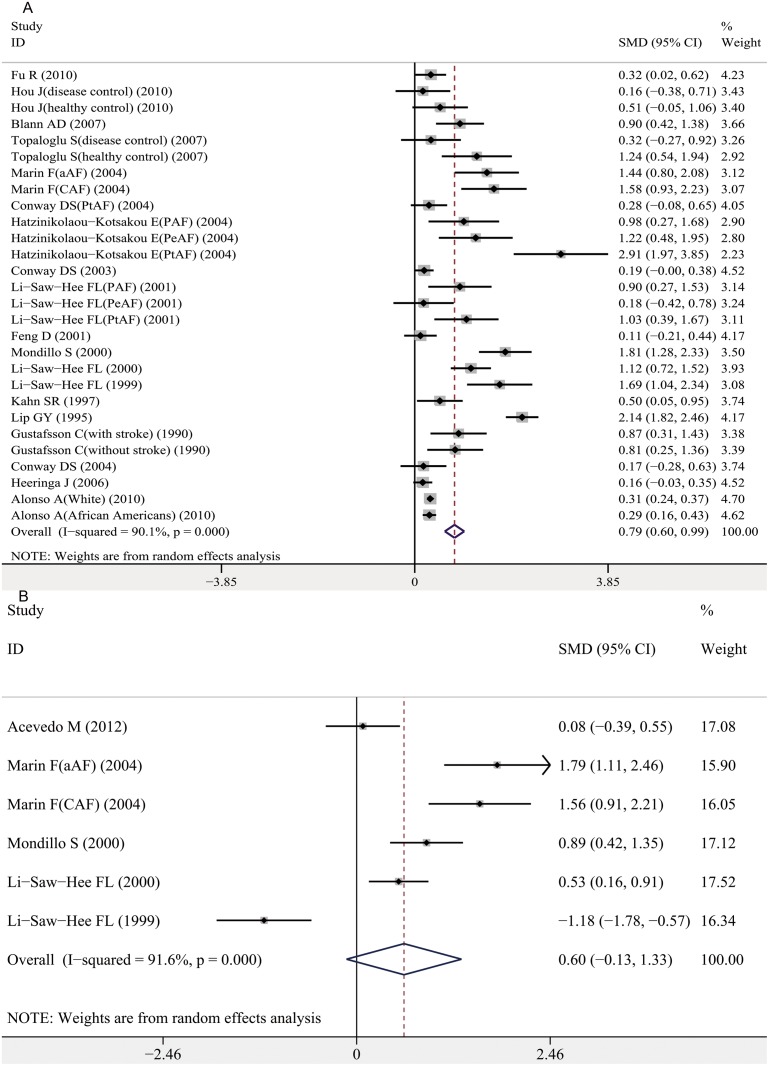
Association between endothelial function markers and AF. A. Von Willebrand factor and AF; B. Soluble thrombomodulin and AF. Forest plots of SMD and overall SMD with 95% CI between AF cases and controls. Black diamonds indicate the SMD, with the size of the square inversely proportional to its variance, and horizontal lines represent the 95% CI. The pooled results are indicated by the black hollow diamond. AF, atrial fibrillation; vWf, von Willebrand factor; sTM, soluble thrombomodulin; PAF, paroxysmal AF; PeAF, persistent AF; PtAF, permanent AF; CAF, chronic AF; aAF, acute AF; SMD, standardized mean difference.

According to a meta-analysis of 19 studies, plasma von Willebrand factor (vWf) was significantly elevated in AF patients compared with controls, with a pooled SMD of 0.79 (95% CI, 0.60–0.99; *P*<0.001) (Fig 5A). No significant association was observed between soluble thrombomodulin (sTM) and AF (pooled SMD 0.60; 95% CI, -0.13–1.33; *P* = 0.107) (Fig 5B). A univariate meta-regression analysis indicated that study design and gender had a significant effect on the level of vWf (S3 Table). However, the test for multivariate model was borderline significant (*P* = 0.0505) (S4 Table). A subgroup analysis determined that the associations between vWf and AF were significant for both case-control and cohort studies (S7 Fig). The results of subgroup analyses after grouping by anticoagulants treatment status were materially consistent with overall effects (S8 Fig).

## Discussion

This meta-analysis summarized the evidence to date regarding the association between a series of hemostatic markers and AF. Our results demonstrated that increased circulating PF-4, BTG, P-selectin, D-dimer, fibrinogen, TAT, F1+2, AT- III, and vWf levels were significantly associated with AF.

Platelet and coagulation activation, fibrinolytic dysfunction, and endothelial damage are four major components of the hemostatic mechanism. Several studies have demonstrated that certain hemostatic markers are associated with AF, but these associations remain controversial [4, 73]. Our meta-analysis determined that circulating PF-4, BTG and P-selectin levels were significantly associated with AF, reflecting an activated platelet state. Coagulation activation markers, such as plasma D-dimer, fibrinogen, TAT, F1+2, and AT- III, were significantly increased in AF patients. Finally, the endothelial dysfunction marker vWf was elevated in AF patients.

The exact mechanism of the association between the abnormal levels of hemostatic markers and AF is unclear. Whether abnormal level of hemostatic markers is a consequence of AF or the presence of alteration in hemostatic markers promotes AF development is still under debate. Accumulative proofs have implied that both mechanisms may interrelate, which means that hemostatic markers are not only a consequence but also a cause. Lip proposed that AF can confer a hypercoagulable state as early as 1995 [74]. Researchers have artificially induced AF in patients and have determined that local cardiac platelet activation could be caused by artificially induced AF [75]. The exact mechanism driving the prothrombotic state in AF is unclear. One of the explanations is that the poorly contractile left atrium in AF leads to increased stasis within the atrium, probably activating multiple hemostatic markers, especially the interaction between erythrocytes and fibrinogen [2]. Several other hypotheses have been proposed, such as inflammation, release of growth factors, abnormal changes in extracellular matrix turnover, and dysfunction in the renin-angiotensin-aldosterone system (RAAS) [3]. The development and maintenance of AF requires an initiating factor (trigger) and an appropriate substrate. Electrophysiological and structural remodeling of the atria provides the substrate [76]. A significant amount of information has demonstrated that inflammation is interrelated with atrial remodeling and related to AF risk [77]. Additionally, inflammation has been associated with endothelial dysfunction, platelet and coagulation activation [78]. The exact mechanism for the interactions between the hemostatic system, inflammation and AF risk needs to be further investigated. Growth factor, in particular vascular endothelial growth factor (VEGF) is able to stimulate tissue factor production which could be one of the driving forces of prothrombotic state in AF [79]. Extracellular matrix turnover plays an important role in atrial remodeling and also may be responsible for prothrombotic state by causing blood stasis and damaging endocardium [3]. Angiotensin II which is a critical factor in RAAS has been shown to increase the release of various proinflammatory cytokines such as interleukin 6 and tumor necrosis factor α [80]. Thus, RAAS may contribute to prothrombotic state through inflammation. On the other hand, the innate alteration in some hemostatic markers at baseline may increase the risk of AF development in the future as suggested by some cohort studies [69–72]. However, the relevant large cohort studies are scarce and a synthetic conclusion is difficult to be drawn.

Additionally, hemostatic markers can be altered by antithrombotic medications. A study has demonstrated that after receiving anticoagulation, the levels of D-dimer and F1+2 in AF patients were lowered [64]. Therefore, we carried out a subgroup analysis by anticoagulants treatment status in our study. We found there was no significant association between MPV, tPA, PAI-1 and AF in participants receiving no anticoagulants and also in studies with different proportion of people anticoagulated in two compared groups. This result was in contrary to overall effects. It indicated anticoagulants had a great impact on these markers and acted as an important confounding factor. One of the main contributors of the abnormal levels of hemostatic markers, namely stasis caused by poorly contractile left atrium in AF may be responsible for the vanishment of association after performing subgroup analysis stratified by anticoagulants treatment status.

AF is sometimes complicated by other cardiovascular diseases, and hemostatic markers are known to be influenced by these comorbidities. Whether hemostatic markers are increased due to AF itself or are coexisting cardiovascular risk factors remains a matter of debate [2]. Feng et al [59] discovered that the association of fibrinogen, vWf, tPA and PAI-1 and AF became nonsignificant after stratifying according to cardiovascular disease status. We tried to explore the influence of comorbidities by taking hypertension, coronary artery disease, cerebrovascular accidents and diabetes mellitus into meta-regression model. However, we only found hypertension and coronary artery disease were significantly associated with level of platelet count.

There is also a strong association between AF and the risk of thromboembolic stroke, although the mechanism for this increased risk has not been fully determined. The pathogenesis is multifactorial and stasis in a poorly contractile left atrium is partially responsible [3]. In addition, some studies determined that MPV, vWf, D-dimer and tPA were able to effectively predict subsequent thromboembolic events in patients with AF [81–83]. Considering the predictive role of these hemostatic markers for thromboembolic events, the inclusion of these markers in thromboembolic risk prediction models for the identification of “high risk” or “true low risk” subjects is worth attempting.

To the best of our knowledge, this is the first systematic and comprehensive meta-analysis examining the associations between a series of hemostatic markers and AF. Due to the currently high incidence of AF and subsequent thromboembolic events, identifying of biological markers that can predict AF risk and hypercoagulable propensity has important clinical implications. However, several limitations of this study should be considered when interpreting our results. High heterogeneity exists among studies. Therefore, we performed a meta-regression analysis to identify potential sources of heterogeneity; gender, type of AF, and comorbidities may explain a portion of this heterogeneity. However, not all of the relevant covariates, such as heart failure and inflammatory factors, are available for each study included in our meta-regression. Therefore, the heterogeneity and unknown confounders may still influence true associations. Secondly, observational studies especially case-control studies could only determine whether there was an excess risk of adverse outcome in association with biomarkers. Verifying causality requires more high-quality, large sample, and more stringent evidence. Our study as a systematic review and meta-analysis summarized the evidences in this field and could provide useful clues and generate initial data for future studies. Finally, statistical tests suggested that there might be publication bias for studies examining BTG, D-dimer, fibrinogen, AT- III and vWf (S9 Fig).

## Conclusion

In conclusion, our meta-analysis demonstrated that elevated circulating hemostatic markers (PF-4, BTG, P-selectin, D-dimer, fibrinogen, TAT, F1+2, AT-III, and vWf) were associated with AF. Our findings provide useful clues and evidences for future study to elucidate the precise mechanism of the prothrombotic state and the role of hemostatic markers in promoting thromboembolism in AF patients.

## Supporting Information

S1 Checklist PRISMA Checklist. (DOC)

S1 Fig Association of platelet count with AF by type of AF. Black diamond indicates the SMD, with the size of the square inversely proportional to its variance, and horizontal lines represent 95% CI. The pooled results are indicated by the black hollow diamond. AF, atrial fibrillation; PAF, paroxysmal AF; PtAF, permanent AF; SMD, standardized mean difference; CI, confidence interval.
(JPG)

S2 Fig Association of mean platelet volume with AF by anticoagulants treatment status. Black diamond indicates the SMD, with the size of the square inversely proportional to its variance, and horizontal lines represent 95% CI. The pooled results are indicated by the black hollow diamond. AF, atrial fibrillation; MPV, mean platelet volume; SMD, standardized mean difference; CI, confidence interval; Subgroup 1, there was significant difference in the proportion of subjects received anticoagulants between AF patients and controls; Subgroup 4, there was no significant difference in the proportion of anticoagulation treatment in both groups (excluding no patient or all patients received anticoagulants in both groups).
(JPG)

S3 Fig Association of D-dimer with AF by type of AF. Black diamond indicates the SMD, with the size of the square inversely proportional to its variance, and horizontal lines represent 95% CI. The pooled results are indicated by the black hollow diamond. AF, atrial fibrillation; PAF, paroxysmal AF; PtAF, permanent AF; SMD, standardized mean difference; CI, confidence interval.
(JPG)

S4 Fig Association of fibrinogen with AF by publication year. Black diamond indicates the SMD, with the size of the square inversely proportional to its variance, and horizontal lines represent 95% CI. The pooled results are indicated by the black hollow diamond. AF, atrial fibrillation; > = 2000, publication year after 2000; <2000, publication year before 2000; SMD, standardized mean difference; CI, confidence interval.
(JPG)

S5 Fig Association of tissue-type plasminogen activator with AF by anticoagulants treatment status. Black diamond indicates the SMD, with the size of the square inversely proportional to its variance, and horizontal lines represent 95% CI. The pooled results are indicated by the black hollow diamond. AF, atrial fibrillation; tPA, tissue-type plasminogen activator; SMD, standardized mean difference; CI, confidence interval; Subgroup 1, there was significant difference in the proportion of subjects received anticoagulants between AF patients and controls; Subgroup 2, no patient received anticoagulants in both groups; Subgroup 5, anticoagulation information was not available in both groups.
(JPG)

S6 Fig Association of plasminogen activator inhibitor-1 with AF by anticoagulants treatment status. Black diamond indicates the SMD, with the size of the square inversely proportional to its variance, and horizontal lines represent 95% CI. The pooled results are indicated by the black hollow diamond. AF, atrial fibrillation; PAI-1, plasminogen activator inhibitor-1; SMD, standardized mean difference; CI, confidence interval; Subgroup 1, there was significant difference in the proportion of subjects received anticoagulants between AF patients and controls; Subgroup 2, no patient received anticoagulants in both groups; Subgroup 5, anticoagulation information was not available in both groups.
(JPG)

S7 Fig Association of vonWillebrand factor with AF by study design. Black diamond indicates the SMD, with the size of the square inversely proportional to its variance, and horizontal lines represent 95% CI. The pooled results are indicated by the black hollow diamond. vWf, vonWillebrand factor; AF, atrial fibrillation; PAF, paroxysmal AF; PeAF, persistent AF: PtAF, permanent AF; aAF, acute AF; CAF, chronic AF; SMD, standardized mean difference; CI, confidence interval.
(JPG)

S8 Fig Association of vonWillebrand factor with AF by anticoagulants treatment status. Black diamond indicates the SMD, with the size of the square inversely proportional to its variance, and horizontal lines represent 95% CI. The pooled results are indicated by the black hollow diamond. AF, atrial fibrillation; PAF, paroxysmal AF; PeAF, persistent AF: PtAF, permanent AF; aAF, acute AF; CAF, chronic AF; SMD, standardized mean difference; CI, confidence interval; Subgroup 1, there was significant difference in the proportion of subjects received anticoagulants between AF patients and controls; Subgroup 2, no patient received anticoagulants in both groups; Subgroup 4, there was no significant difference in the proportion of anticoagulation treatment in both groups; Subgroup 5, anticoagulation information was not available in both groups.
(JPG)

S9 Fig Funnel plot analysis to detect publication bias. Each point represents a separate study for the indicated association. *P* values were calculated by Begg’s test. **A.** Platelet count and AF (*P* = 0.484); **B.** MPV and AF (*P* = 0.462); **C.** PF-4 and AF (*P* = 0.584); **D.** BTG and AF (*P* = 0.042); **E.** P-selectin and AF (*P* = 0.535); **F.** D-dimer and AF (*P* = 0.001); **G.** Fibrinogen and AF (*P*<0.001); **H.** TAT and AF (*P* = 1.000); **I.** F1+2 and AF (*P* = 0.466); **J.** AT-III and AF (*P* = 0.024); **K.** tPA and AF (*P* = 1.000); **L.** PAI-1 and AF (*P* = 0.661); **M.** vWf and AF (*P*<0.001); **N.** sTM and AF (*P* = 0.452).
(DOC)

S1 Table Quality assessment score scale. (DOC)

S2 Table Characteristics of studies included for meta-analysis of association between haemostatic markers and AF. (DOC)

S3 Table Univariate meta-regression results for haemostatic markers. (DOC)

S4 Table Multivariate meta-regression results for fibrinogen and vWf. (DOC)
