# Supplementary figures and images for: Association of Hemostatic Markers with Atrial Fibrillation: A Meta-Analysis and Meta-Regression
Source: PLoS One. 2015 Apr 17;10(4):e0124716. doi: 10.1371/journal.pone.0124716 (PMC4401562; doi:10.1371/journal.pone.0124716)

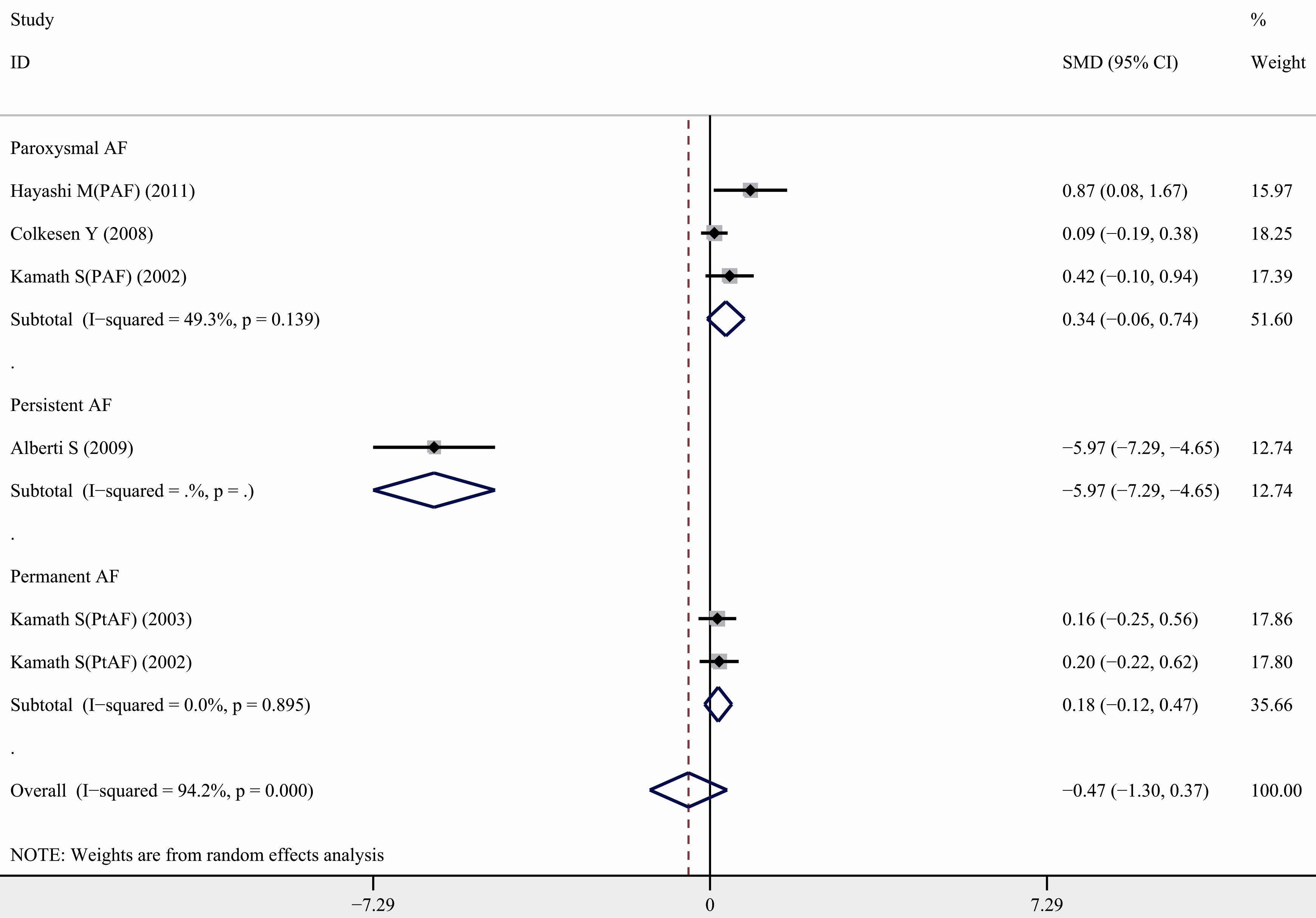

Supplement: S1 Fig — Black diamond indicates the SMD, with the size of the square inversely proportional to its variance, and horizontal lines represent 95% CI. The pooled results are indicated by the black hollow diamond. AF, atrial fibrillation; PAF, paroxysmal AF; PtAF, permanent AF; SMD, standardized mean difference; CI, confidence interval. (JPG) [file pone.0124716.s002.jpg]

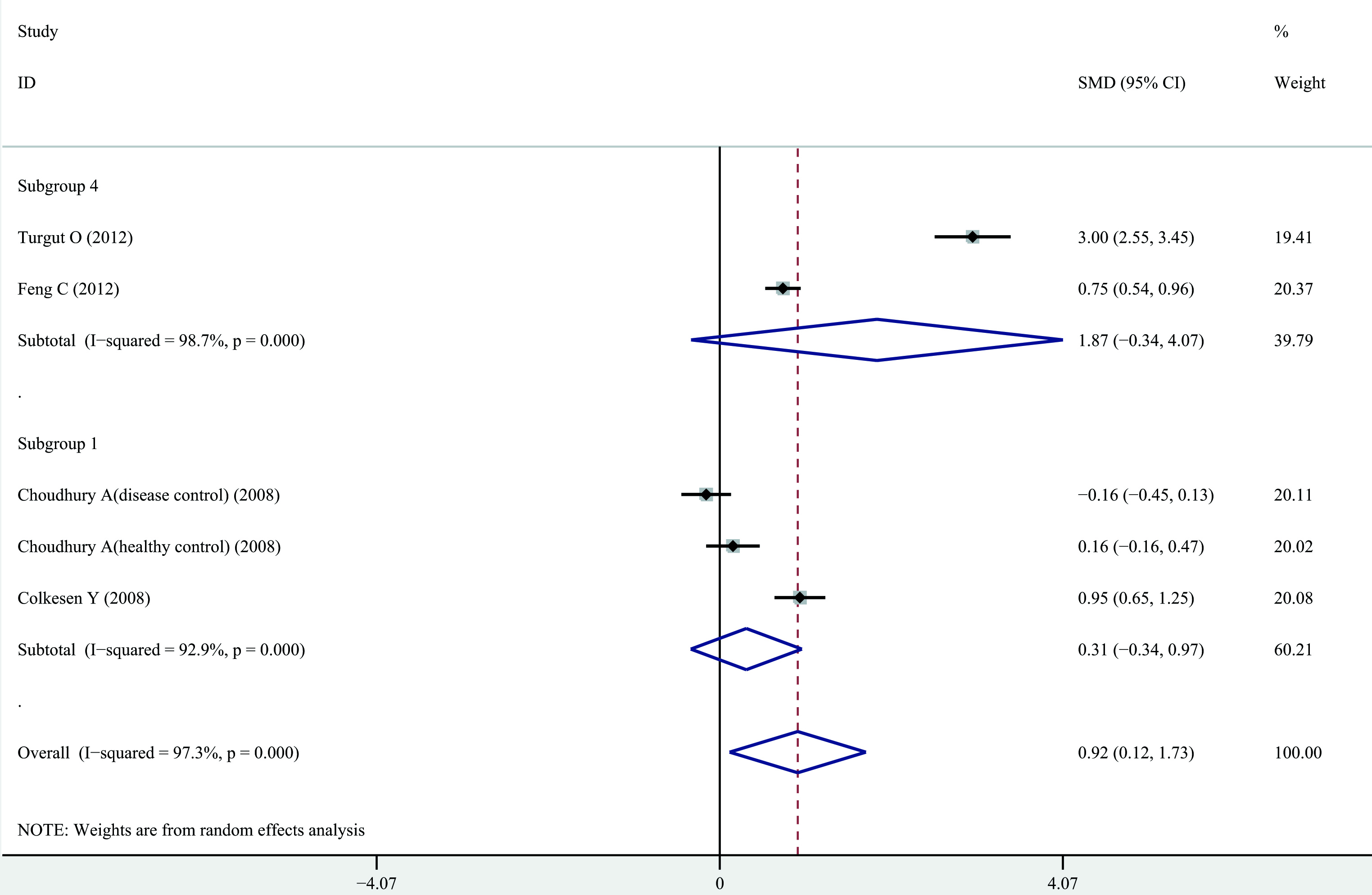

Supplement: S2 Fig — Black diamond indicates the SMD, with the size of the square inversely proportional to its variance, and horizontal lines represent 95% CI. The pooled results are indicated by the black hollow diamond. AF, atrial fibrillation; MPV, mean platelet volume; SMD, standardized mean difference; CI, confidence interval; Subgroup 1, there was significant difference in the proportion of subjects received anticoagulants between AF patients and controls; Subgroup 4, there was no significant difference in the proportion of anticoagulation treatment in both groups (excluding no patient or all patients received anticoagulants in both groups). (JPG) [file pone.0124716.s003.jpg]

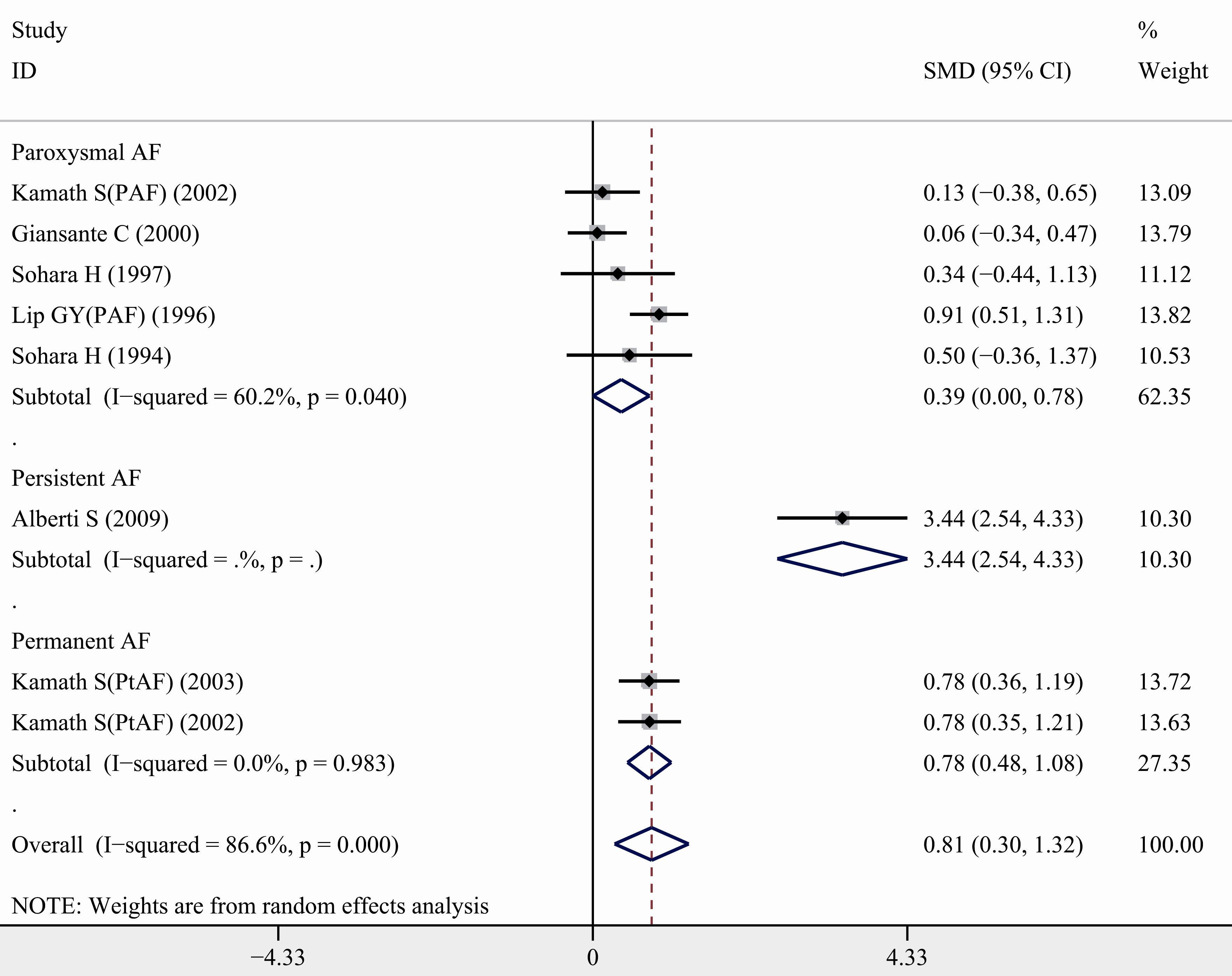

Supplement: S3 Fig — Black diamond indicates the SMD, with the size of the square inversely proportional to its variance, and horizontal lines represent 95% CI. The pooled results are indicated by the black hollow diamond. AF, atrial fibrillation; PAF, paroxysmal AF; PtAF, permanent AF; SMD, standardized mean difference; CI, confidence interval. (JPG) [file pone.0124716.s004.jpg]

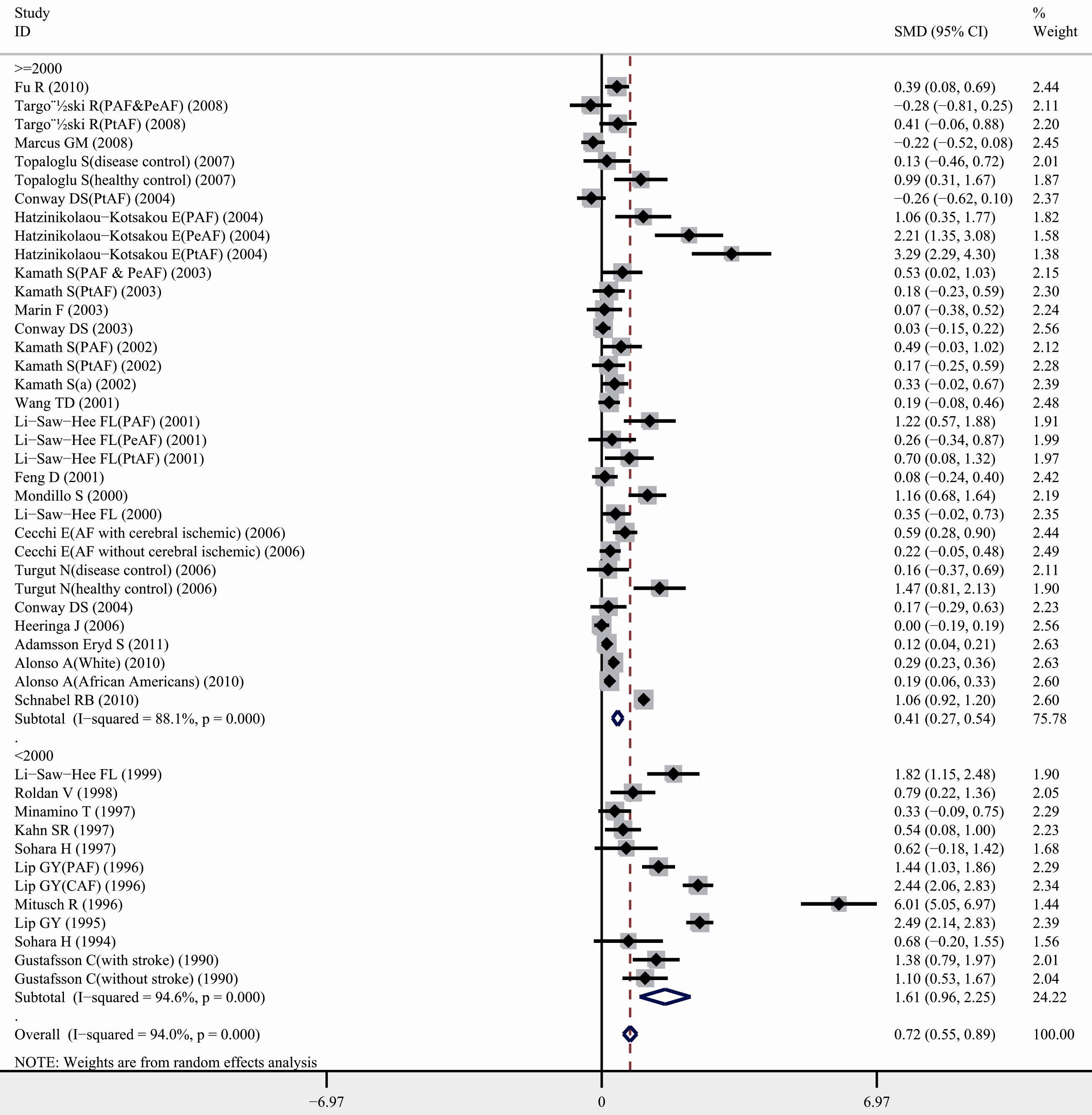

Supplement: S4 Fig — Black diamond indicates the SMD, with the size of the square inversely proportional to its variance, and horizontal lines represent 95% CI. The pooled results are indicated by the black hollow diamond. AF, atrial fibrillation; > = 2000, publication year after 2000; <2000, publication year before 2000; SMD, standardized mean difference; CI, confidence interval. (JPG) [file pone.0124716.s005.jpg]

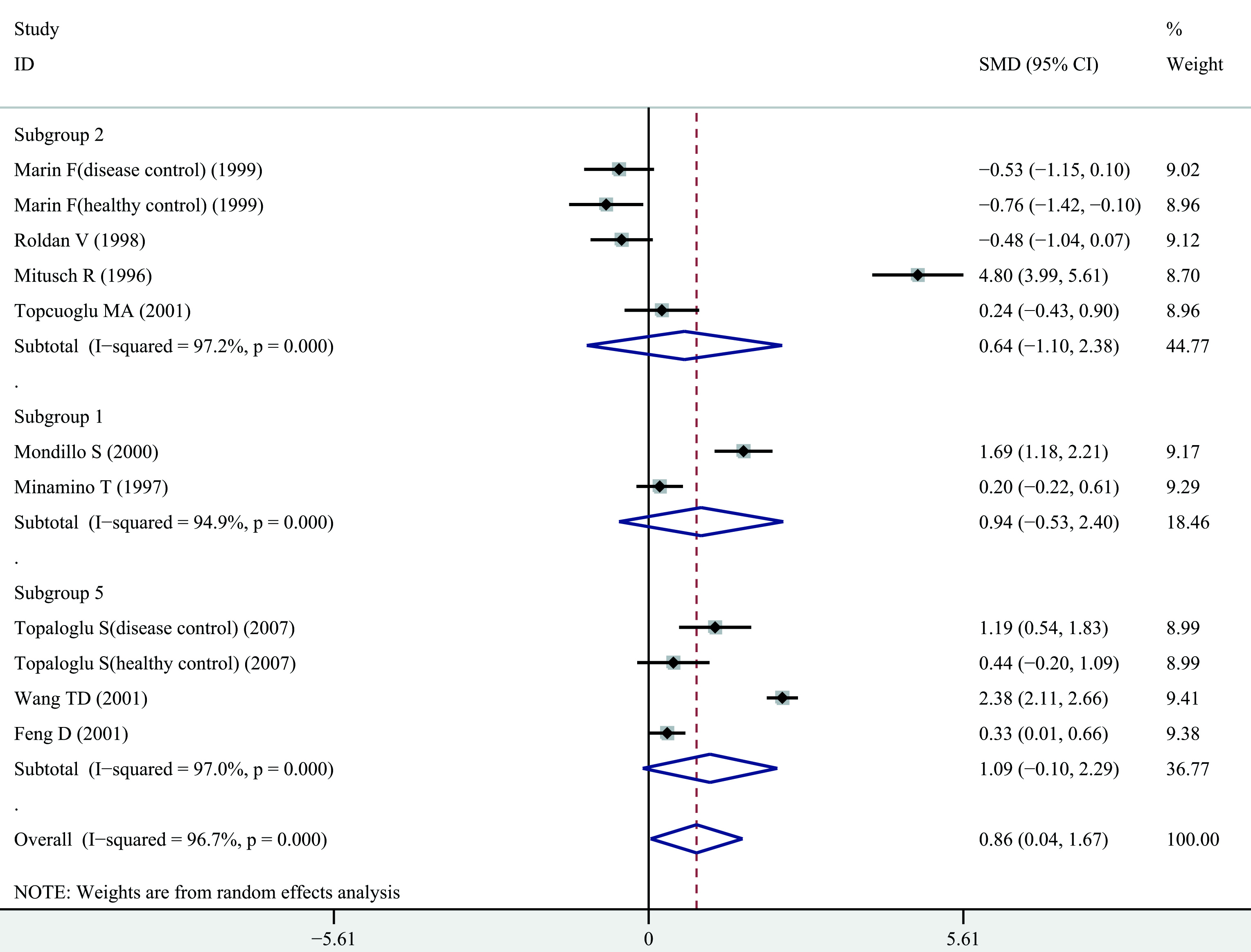

Supplement: S5 Fig — Black diamond indicates the SMD, with the size of the square inversely proportional to its variance, and horizontal lines represent 95% CI. The pooled results are indicated by the black hollow diamond. AF, atrial fibrillation; tPA, tissue-type plasminogen activator; SMD, standardized mean difference; CI, confidence interval; Subgroup 1, there was significant difference in the proportion of subjects received anticoagulants between AF patients and controls; Subgroup 2, no patient received anticoagulants in both groups; Subgroup 5, anticoagulation information was not available in both groups. (JPG) [file pone.0124716.s006.jpg]

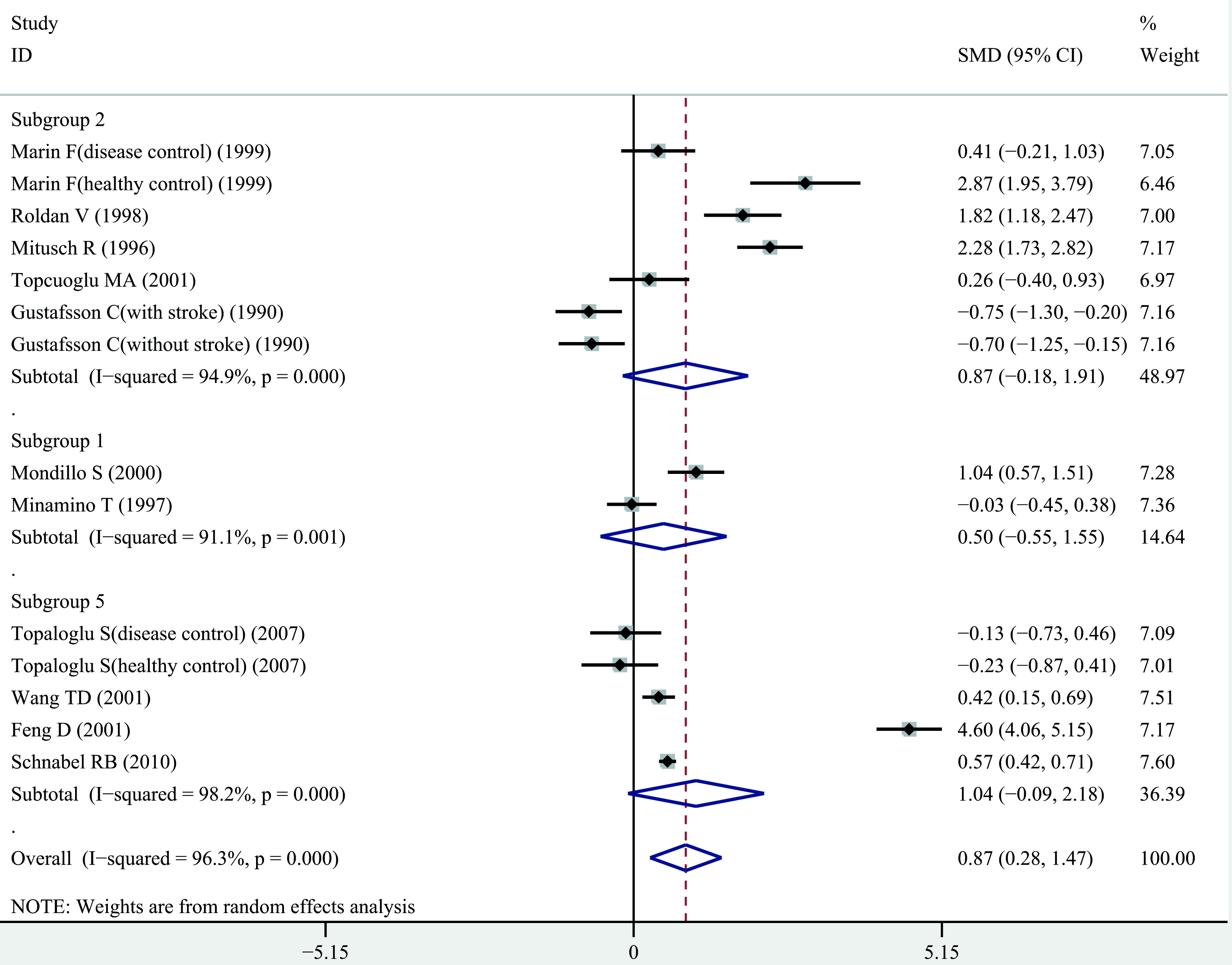

Supplement: S6 Fig — Black diamond indicates the SMD, with the size of the square inversely proportional to its variance, and horizontal lines represent 95% CI. The pooled results are indicated by the black hollow diamond. AF, atrial fibrillation; PAI-1, plasminogen activator inhibitor-1; SMD, standardized mean difference; CI, confidence interval; Subgroup 1, there was significant difference in the proportion of subjects received anticoagulants between AF patients and controls; Subgroup 2, no patient received anticoagulants in both groups; Subgroup 5, anticoagulation information was not available in both groups. (JPG) [file pone.0124716.s007.jpg]

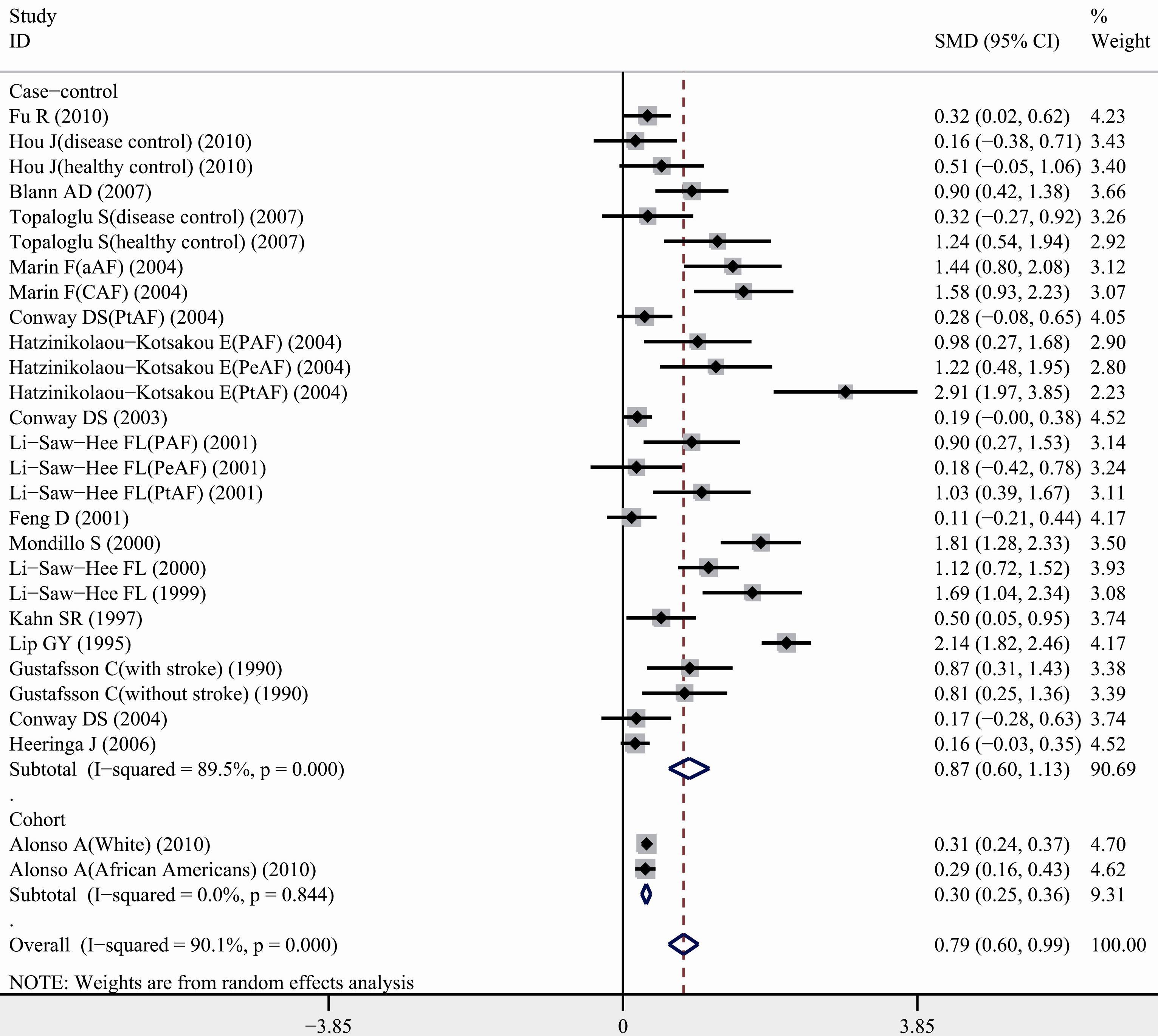

Supplement: S7 Fig — Black diamond indicates the SMD, with the size of the square inversely proportional to its variance, and horizontal lines represent 95% CI. The pooled results are indicated by the black hollow diamond. vWf, vonWillebrand factor; AF, atrial fibrillation; PAF, paroxysmal AF; PeAF, persistent AF: PtAF, permanent AF; aAF, acute AF; CAF, chronic AF; SMD, standardized mean difference; CI, confidence interval. (JPG) [file pone.0124716.s008.jpg]

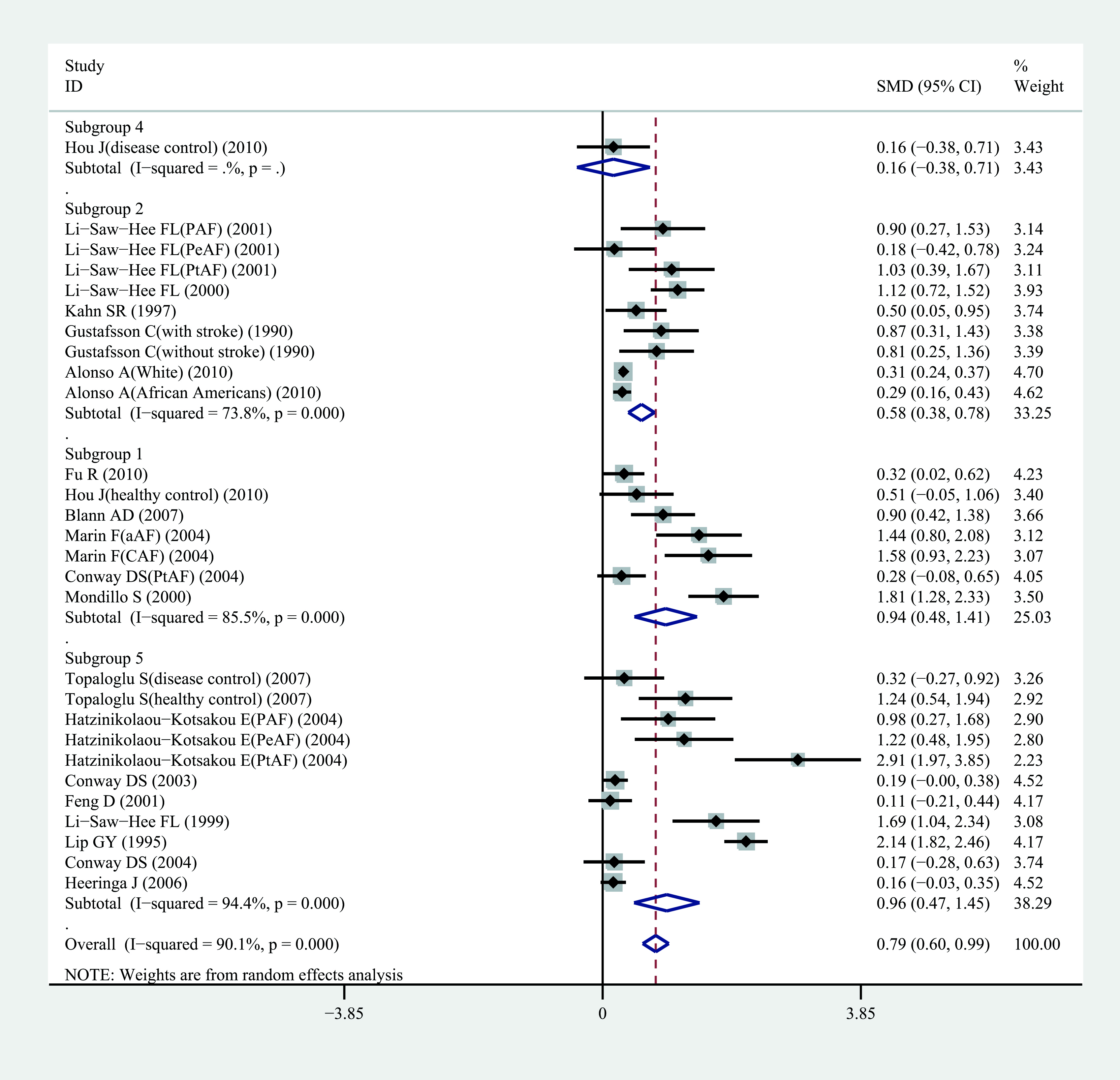

Supplement: S8 Fig — Black diamond indicates the SMD, with the size of the square inversely proportional to its variance, and horizontal lines represent 95% CI. The pooled results are indicated by the black hollow diamond. AF, atrial fibrillation; PAF, paroxysmal AF; PeAF, persistent AF: PtAF, permanent AF; aAF, acute AF; CAF, chronic AF; SMD, standardized mean difference; CI, confidence interval; Subgroup 1, there was significant difference in the proportion of subjects received anticoagulants between AF patients and controls; Subgroup 2, no patient received anticoagulants in both groups; Subgroup 4, there was no significant difference in the proportion of anticoagulation treatment in both groups; Subgroup 5, anticoagulation information was not available in both groups. (JPG) [file pone.0124716.s009.jpg]
