# Supplementary material for: Association of Hemostatic Markers with Atrial Fibrillation: A Meta-Analysis and Meta-Regression
Source: PLoS One. 2015 Apr 17;10(4):e0124716. doi: 10.1371/journal.pone.0124716 (PMC4401562; doi:10.1371/journal.pone.0124716)
Supplement: S9 Fig — Each point represents a separate study for the indicated association. P values were calculated by Begg’s test. A. Platelet count and AF (P = 0.484); B. MPV and AF (P = 0.462); C. PF-4 and AF (P = 0.584); D. BTG and AF (P = 0.042); E. P-selectin and AF (P = 0.535); F. D-dimer and AF (P = 0.001); G. Fibrinogen and AF (P<0.001); H. TAT and AF (P = 1.000); I. F1+2 and AF (P = 0.466); J. AT-III and AF (P = 0.024); K. tPA and AF (P = 1.000); L. PAI-1 and AF (P = 0.661); M. vWf and AF (P<0.001); N. sTM and AF (P = 0.452). (DOC) [file pone.0124716.s010.doc]

**A. B.**

**
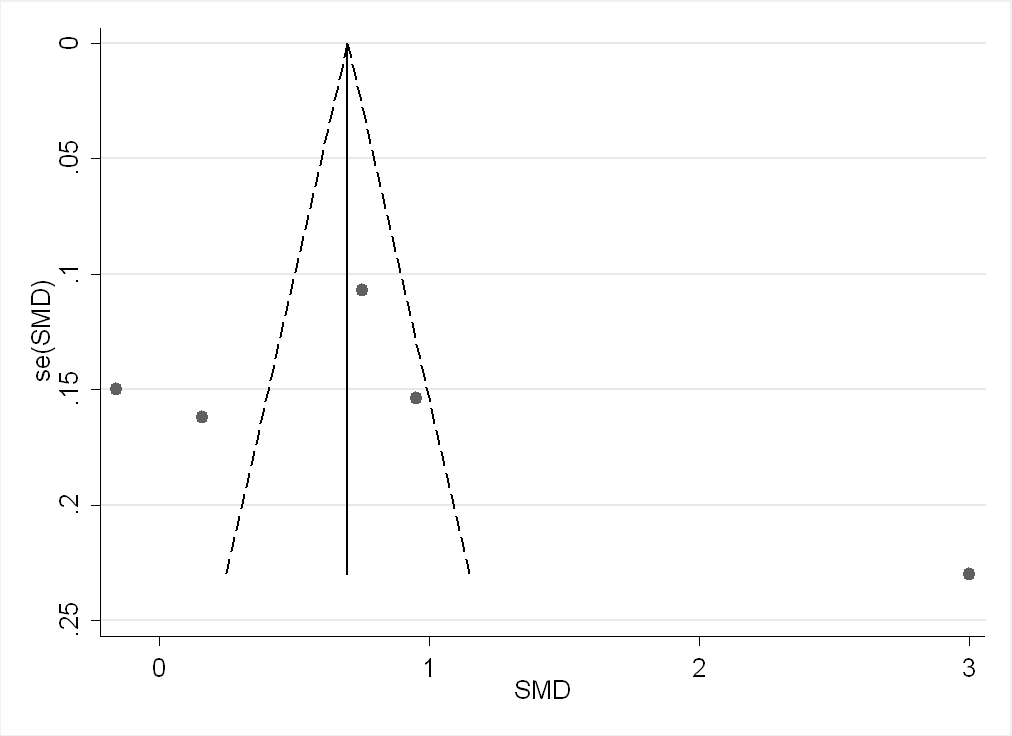

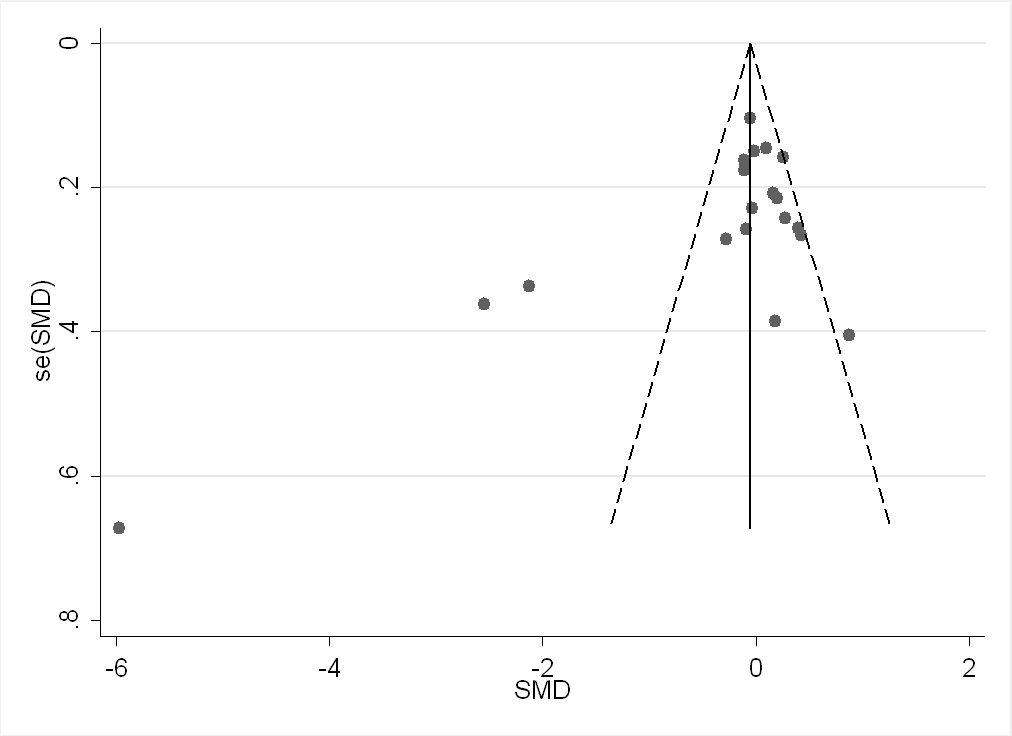
**


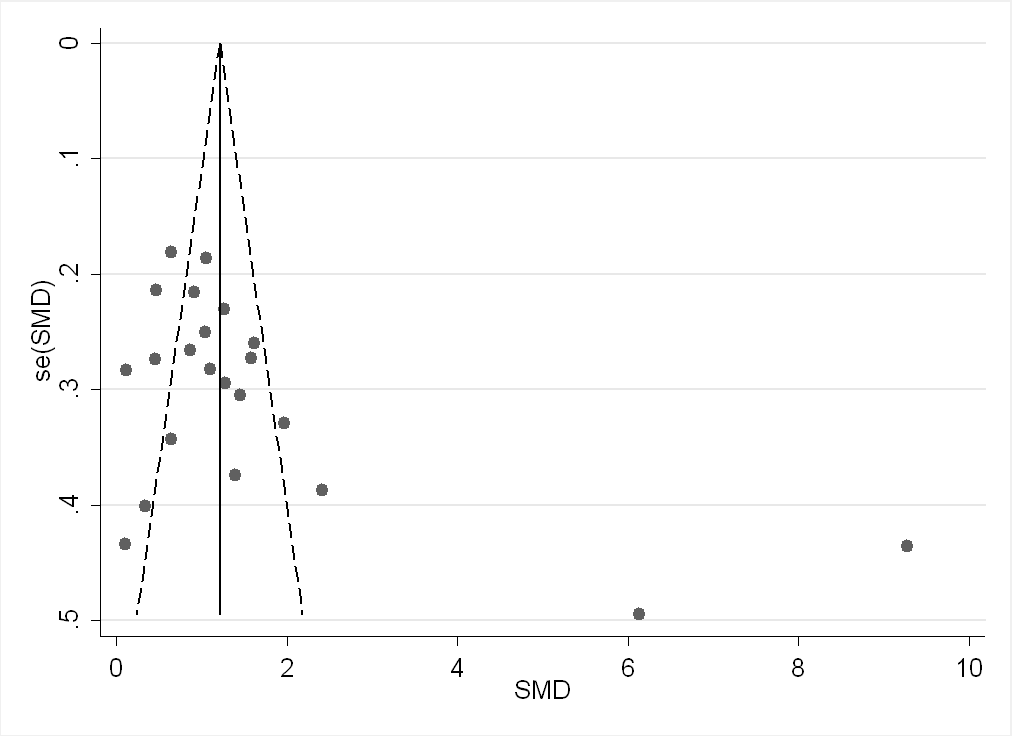
**C. D.**

**
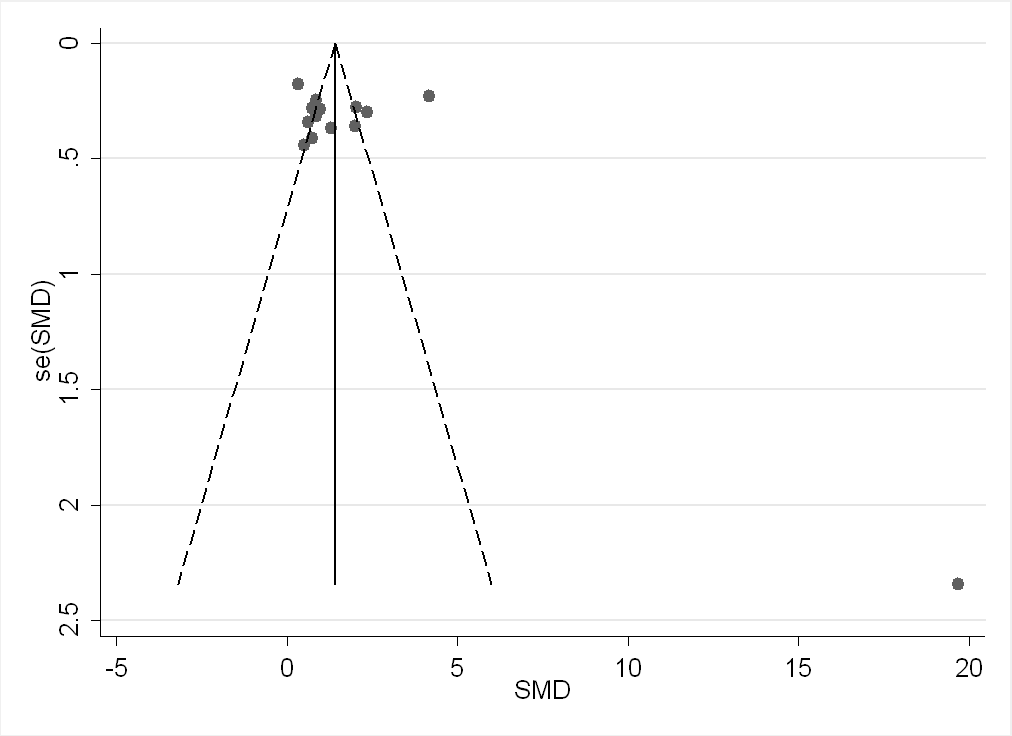
**

**E. F.**

**
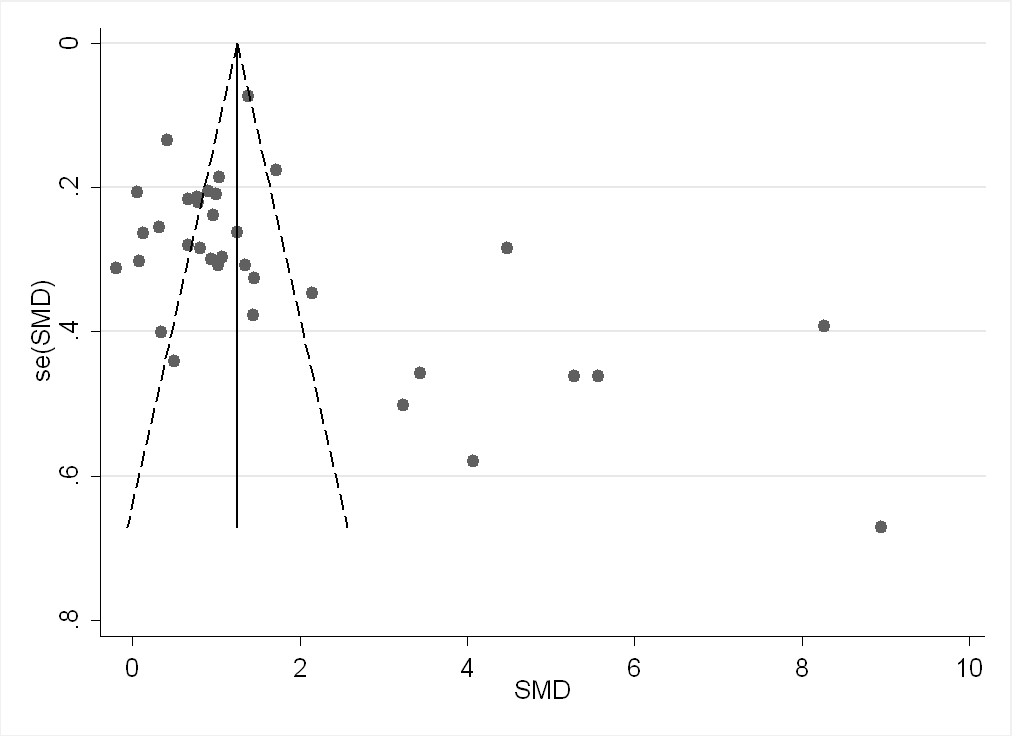

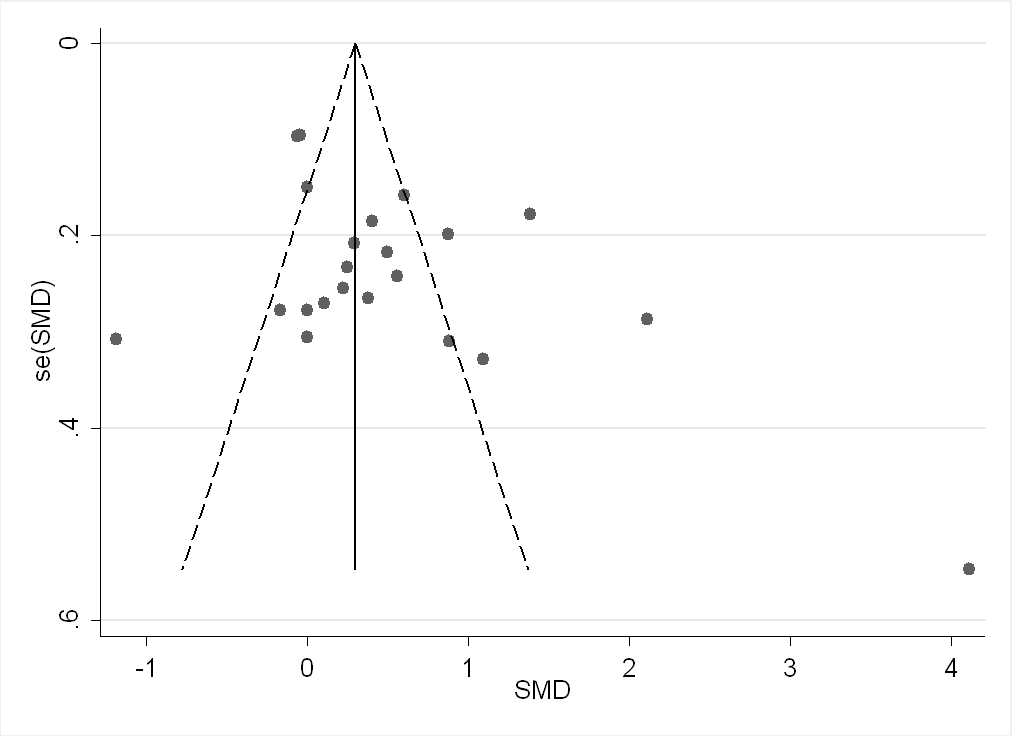
**

**G. H.**

**
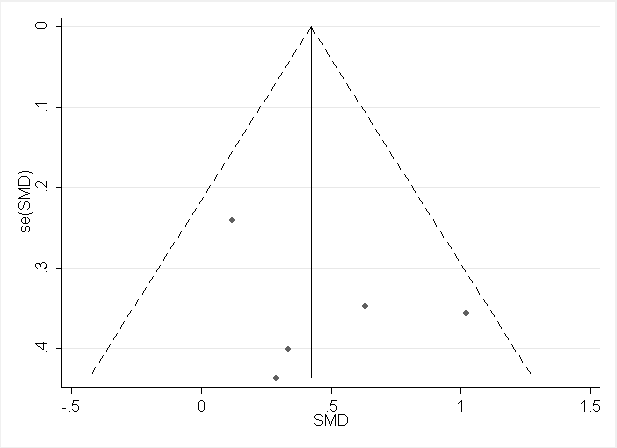

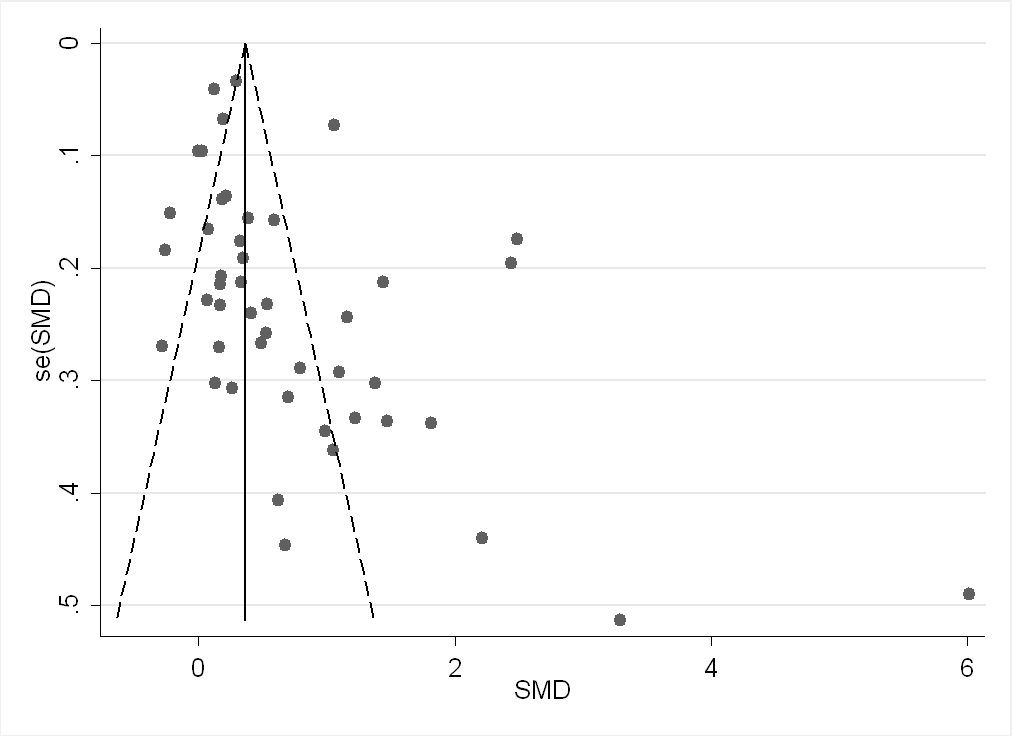
**

**I. J.**


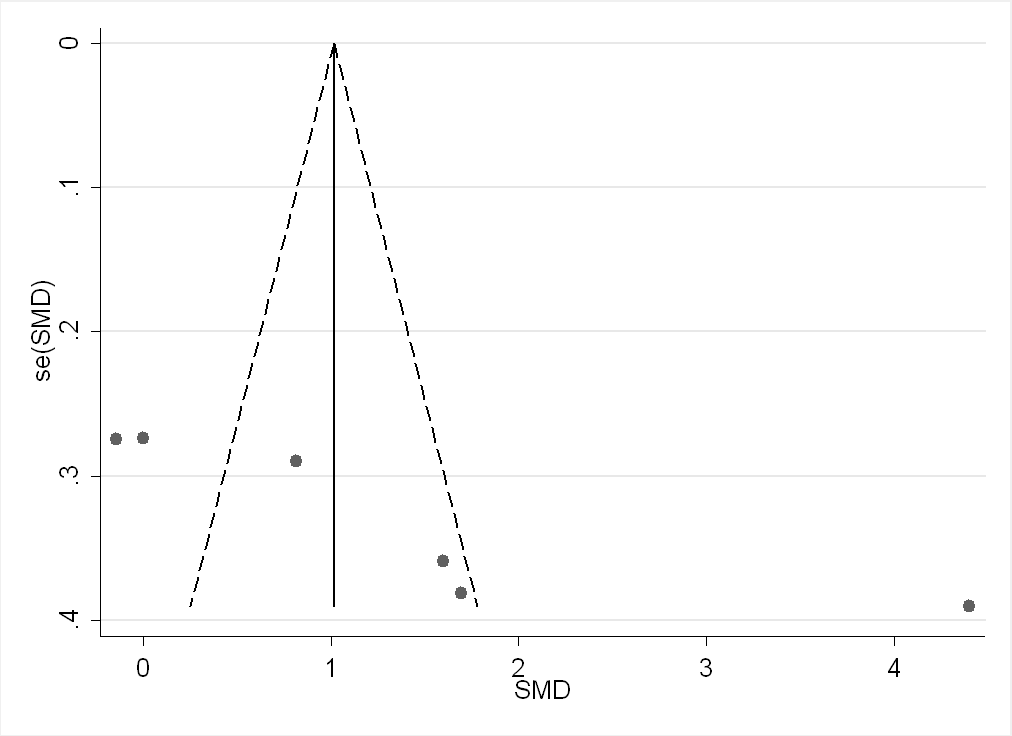
**
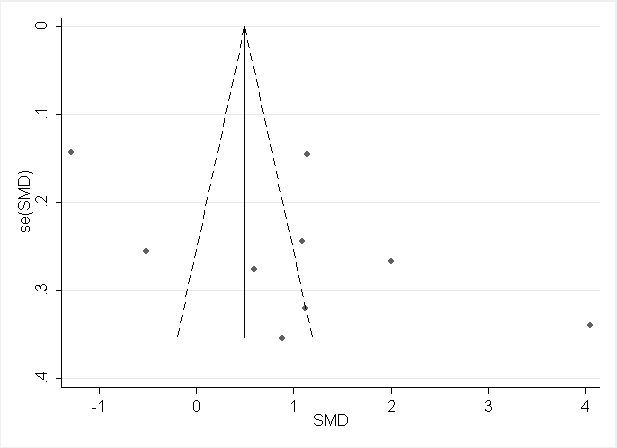
**

**K. L.**

**
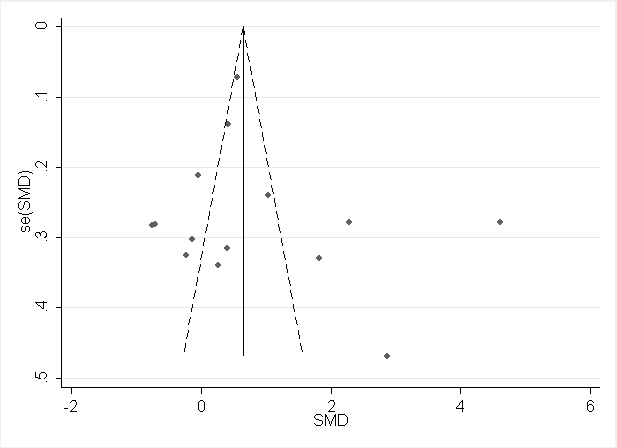

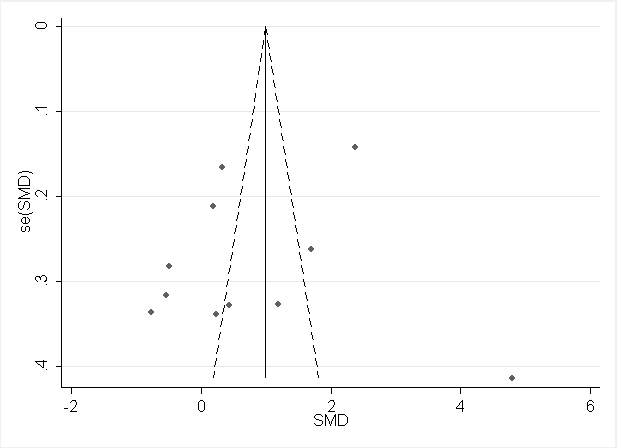
**

**M. N.**

**
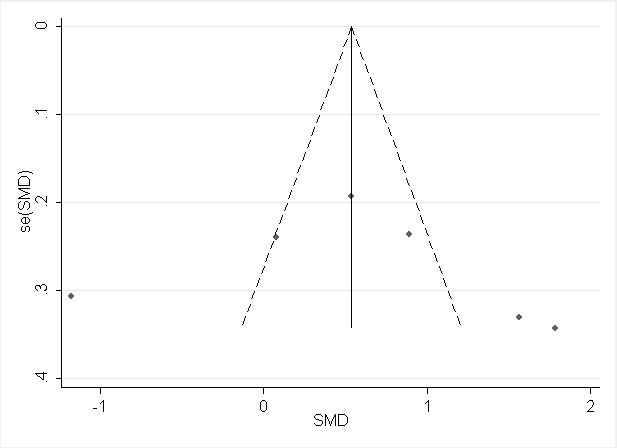

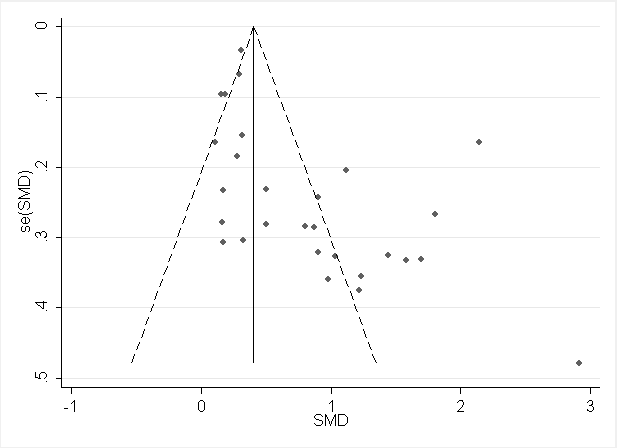
**

**Supplementary Figure 8**. Funnel plot analysis to detect publication bias. Each point represents a separate study for the indicated association. *P* values were calculated by Begg’s test. **A.** Platelet count and AF (*P*=0.484); **B**. MPV and AF (*P*=0.462); **C.** PF-4 and AF (*P*=0.584); **D.** BTG and AF (*P*=0.042); **E.** P-selectin and AF (*P*=0.535); **F.** D-dimer and AF (*P*=0.001); **G.** Fibrinogen and AF (*P*<0.001); **H.** TAT and AF (*P*=1.000); **I.** F1+2 and AF (*P*=0.466); **J.** AT-III and AF (*P*=0.024); **K.** tPA and AF (*P*=1.000); **L**. PAI-1 and AF (*P*=0.661); **M.** vWf and AF (*P*<0.001); **N.** sTM and AF (*P*=0.452).
