# Supplementary material for: Association of Hemostatic Markers with Atrial Fibrillation: A Meta-Analysis and Meta-Regression
Source: PLoS One. 2015 Apr 17;10(4):e0124716. doi: 10.1371/journal.pone.0124716 (PMC4401562; doi:10.1371/journal.pone.0124716)
Supplement: S2 Table — (DOC) [file pone.0124716.s012.doc]

**Table S2. Characteristics of studies included for meta-analysis of association between haemostatic markers and AF**

| First author | Year | Country/  region | Study design | Number of  participants | | Classification  of AF | Mean age | | Male  (%) | | Anticoagulants use (%) | | | | Haemostatic Markers | NOS  score |
| --- | --- | --- | --- | --- | --- | --- | --- | --- | --- | --- | --- | --- | --- | --- | --- | --- |
|  |  |  |  | Case | Control |  | Case | Control | Case | Control | Case | Control | | |  |  |
| Turgut O | 2012 | Turkey | Case-control | 81 | 81 | AF | 64.0 | 62.0 | 50.6 | 53.1 | 28.0 | 20.0 | | | PC , MPV | 7 |
| Feng C | 2012 | China | Case-control | 185 | 189 | AF | 65.9 | 65.7 | 62.7 | 60.8 | 76.8 | 83.1 | | | PC , MPV | 8 |
| Acevedo M | 2012 | Chile | Case-control | 130 | 20 | Paroxysmal or  persistent AF | 67.0 | - | 53.8 | - | 0 | 0 | | | sTM, TAT, P-sel, | 8 |
| Hayashi M | 2011 | Japan | Case-control | 14 | 13 | Paroxysmal AF | 53.1 | 62.8 | 92.9 | 92.3 | 100 | 100 | | | PC | 7 |
|  |  |  |  | 14 | - | Chronic AF | 60.1 | - | 92.9 | - | 100 | 100 | | | PC |  |
| Zorlu A | 2011 | Turkey | Cohort | 31 | 119 | AF | 72.0* | 67.0 | 64.5 | 59.7 | 0 | 0 | | | D-dimer | 8 |
| Adamsson Eryd S | 2011 | Sweden | Cohort | 667 | 5364 | AF | 47.8* | 46.7 | 100 | 100 | - | - | | | Fib | 9 |
| Fu R | 2010 | China | Case-control | 90 | 79 | AF | 54.1 | 54.8 | 70.0 | 57.0 | 22.0* | 0 | | | P-sel, Fib, vWf | 8 |
| Hou J | 2010 | China | Case-control | 26 | 26 | AF  (disease control) | 65.2 | 64.5 | 57.7 | 57.7 | 7.7 | 11.5 | | | P-sel, vWf,D-dimer | 8 |
|  |  |  |  | 26 | 26 | AF  (healthy control) | 65.2 | 64.5 | 57.7 | 57.7 | 7.7* | 0 | | | P-sel,vWf,D-dimer |  |
| Alonso A | 2010 | USA | Cohort | 976 | 10131 | AF (White) | 57.3 | 54.1 | 58.5 | 46.1 | 0 | 0 | | | Fib, vWf | 9 |
|  |  |  |  | 233 | 3518 | AF (African-American) | 56.2 | 53.4 | 44.6 | 37.8 | 0 | 0 | | | Fib, vWf |  |
| Schnabel RB | 2010 | USA | Cohort | 209 | 2911 | AF | 66.3 | 57.8 | 59.8 | 44.8 | - | - | | | Fib, D-dimer, PAI | 9 |
| Alberti S | 2009 | Italy | Case-control | 17 | 34 | Persistent AF | 68.1 | 60.8 | 47.1 | 47.1 | 0 | 0 | | | Platelet, P-sel,  D-dimer | 7 |
| Gartner W | 2008 | Austria | Case-control | 222 | 28 | AF | 64.5 | 54.4 | 63.5 | 67.9 | 55 | 50 | | | D-dimer | 6 |
| Targoński R | 2008 | Poland | Case-control | 26 | 30 | Paroxymal and  persistent AF | 70.3 | 68.7 | 65.4 | 70.0 | - | - | | | Fib | 8 |
|  |  |  |  | 43 | 71 | Permanent AF | 69.9 | 68.7 | 62.8 | 70.0 | - | - | | | Fib |  |
| Choudhury A | 2008 | UK | Case-control | 121 | 71 | AF (disease control) | 62.6 | 64.0 | 75.2 | 71.8 | 37.2* | 47.4 | | | PC, MPV, P-sel | 6 |
|  |  |  |  | 121 | 56 | AF (healthy control) | 62.6 | 62.0 | 75.2 | 67.9 | 37.2* | 0 | | | PC, MPV, P-sel |  |
| Colkesen Y | 2008 | Turkey | Case-control | 103 | 87 | Paroxysmal AF | 63.0* | 45.0 | 55.3* | 21.8 | 50.0* | 14.0 | | | PC, MPV, | 8 |
| Marcus GM | 2008 | USA | Case-control | 46 | 925 | AF | 74.0* | 66.0 | 93.5* | 81.1 | - | - | | | Fib | 9 |
| Blann AD | 2007 | UK | Case-control | 54 | 28 | AF | 65.0 | 64.0 | 64.8 | 60.7 | 60.0* | 0 | | | P-sel, vWf | 6 |
| Topaloglu S | 2007 | Turkey | Case-control | 18 | 28 | AF  (disease control) | 37.0 | 32.0 | - | - | - | - | | | Fib, vWf, D-dimer, tPA, PAI. PF-4 | 6 |
|  |  |  |  | 18 | 20 | AF  (healthy control) | 37.0 | 35.0 | - | - | - | - | | | Fib, vWf, D-dimer, tPA, PAI. PF-4 |  |
| Yip HK | 2006 | Taiwan | Case-control | 62 | 20 | AF | 66.2 | 65.3 | 66.1 | 60.0 | 36.0* | 0 | | | PC | 7 |
| Cecchi E | 2006 | Italy | Case-control | 62 | 130 | AF with cerebral ischemic | 75.0 | 72.0 | 61.3 | 59.2 | 100* | 0 | | | Fib | 6 |
|  |  |  |  | 94 | 130 | AF without  cerebral ischemic | 74.0 | 72.0 | 59.6 | 59.2 | 100* | 0 | | | Fib |  |
| Turgut N | 2006 | Turkey | Case-control | 26 | 29 | AF  (disease control) | 67.4 | 64.9 | 30.8 | 58.6 | 38.5* | 20.7 | | | Fib, F1+2 | 8 |
|  |  |  |  | 26 | 20 | AF  (healthy control) | 67.4 | 65.6 | 30.8 | 60.0 | 38.5* | 0 | | | Fib, F1+2 |  |
| Heeringa J | 2006 | UK | Cohort | 162 | 324 | AF | 78.0* | 77.0* | 51.2 | 50.9 | - | - | | | P-sel, Fib, vWf | 8 |
| Roldan V | 2005 | Spain | Case-control | 191 | 74 | AF | 72.0 | - | 51.3 | - | 100* | 62.2 | | | F1+2 | 7 |
| Marin F | 2004 | Spain | Case-control | 24 | 24 | Acute AF | 64.0 | 63.0 | 50.0 | 50.0 | 4.0* | 0 | | | sTM, vWf,  D-dimer | 8 |
|  |  |  |  | 24 | 24 | Chronic AF | 64.0 | 63.0 | 45.8 | 50.0 | 10.0* | 0 | | | sTM, vWf,  D-dimer |  |
| Inoue H | 2004 | Japan | Case-control | 159 | 92 | AF with  comorbidities | - | - | - | - | - | - | | | D-dimer, F1+2,  F-4, BTG | 7 |
|  |  |  |  | 87 | 19 | Lone AF | - | - | - | - | - | - | | | D-dimer, F1+2,  F-4, BTG |  |
| Conway DS | 2004 | UK | Case-control | 106 | 41 | Permanent AF | 69.0 | 67.0 | 63.2 | 61.0 | 86.0* | 0 | | | P-sel. Fib, vWf | 6 |
| Hatzinikolaou-Kotsakou E | 2004 | Greece | Case-control | 18 | 17 | Paroxysmal AF | 59.0 | 59.0 | 72.2 | 82.4 | - | - | | | Fib, vWf | 8 |
|  |  |  |  | 17 | 17 | Persistent AF | 61.0 | 59.0 | 64.7 | 82.4 | - | - | | | Fib, vWf |  |
|  |  |  |  | 20 | 17 | Permanent AF | 64.0 | 59.0 | 70.0 | 82.4 | - | - | | | Fib, vWf |  |
| Atalar E | 2004 | Turkey | Case-control | 15 | 22 | Paroxysmal AF | 45.0 | 47.0 | 60.0 | 63.6 | 0 | 0 | | | PF-4, BTG | 6 |
|  |  |  |  | 25 | 22 | Permanent AF | 51.0 | 47.0 | 64.0 | 63.6 | 0 | 0 | | | PF-4, BTG |  |
| Conway DS | 2004 | Turkey | Case-control | 37 | 37 | Persistent AF | 67.0 | 68.0 | 73.0 | 67.6 | - | - | | | P-sel, Fib, vWf, | 6 |
| Kamath S | 2003 | UK | Case-control | 31 | 31 | Paroxysmal and  persistent AF | 61.0 | 66.0 | 61.3 | 41.9 | 0 | 0 | | | PC, P-sel, Fib,  D-dimer, BTG | 6 |
|  |  |  |  | 93 | 31 | Permanent AF | 66.0 | 66.0 | 63.4 | 41.9 | 0 | 0 | | | PC, P-sel, Fib,  D-dimer, BTG |  |
| Marin F | 2003 | Spain | Case-control | 48 | 32 | Chronic AF | 71.0 | 70.0 | 62.5 | 46.9 | 38.0* | 9.0 | | | Fib, F1+2 | 7 |
| Conway DS | 2003 | UK | Cohort | 162 | 324 | AF | 78.0 | 77.0 | 51.2 | 50.9 | - | - | | | P-sel, Fib, vWf | 8 |
| Kamath S | 2002 | UK | Case-control | 29 | 29 | Paroxysmal AF | 61.0 | 65.0 | 55.2 | 41.4 | 37.9* | 0 | | | PC, P-sel, Fib,  D-dimer, BTG | 7 |
|  |  |  |  | 87 | 29 | Permanent AF | 65.0 | 65.0 | 63.2 | 41.4 | 37.9* | 0 | | | PC, P-sel, Fib,  D-dimer, BTG |  |
| Kamath S | 2002 | UK | Case-control | 93 | 50 | AF | 70.0 | 70.0 | 62.4 | 46.0 | 0 | 0 | | | PC,Fib, D-dimer,  BTG | 6 |
| Kamath S | 2002 | UK | Case-control | 34 | 23 | AF | 73.0 | - | 50.0 | - | 0 | 0 | | | PC, P-sel, BTG | 6 |
| Wang TD | 2001 | Taiwan | Cohort | 53 | 3159 | AF | 66.1* | 53.9 | 56.6 | 46.7 | - | - | | | Fib, tPA, PAI | 9 |
| Li-Saw-Hee FL | 2001 | UK | Case-control | 23 | 20 | Paroxysmal AF | 65.0 | 63.0 | 69.6 | 85.0 | 69.6 | 0 | | | P-sel, Fib, vWf | 8 |
|  |  |  |  | 23 | 20 | Persistent AF | 65.0 | 63.0 | 69.6 | 85.0 | 100 | 0 | | | P-sel, Fib, vWf |  |
|  |  |  |  | 23 | 20 | Permanent AF | 67.0 | 63.0 | 69.6 | 85.0 | 100 | 0 | | | P-sel, Fib, vWf |  |
| Feng D | 2001 | USA | Case-control | 47 | 167 | AF | 62.0 | 62.3 | 74.5 | 72.5 | 76.6 | - | | | Fib, vWf, tPA, PAI | 8 |
| Topcuoglu MA | 2001 | Turkey | Case-control | 15 | 21 | Lone AF | 61.9 | 62.8 | 66.7 | 57.1 | 0 | 0 | | | TAT, tPA, PAI,  F1+2 | 6 |
| Mondillo S | 2000 | Italy | Case-control | 45 | 35 | AF | 67.6 | 66.3 | 80.0 | 85.7 | 55.5* | 0 | | | sTM, Fib, D-dimer, vWf, tPA, PAI,  PF-4, BTG | 7 |
| Giansante C | 2000 | Italy | Case-control | 35 | 70 | Paroxysmal AF | 64.0 | 63.0 | 54.3 | 57.1 | 0 | | 0 | | D-dimer | 7 |
| Li-Saw-Hee FL | 2000 | UK | Case-control | 52 | 60 | AF | 68.0 | 66.0 | 80.8 | 75.0 | 0 | | | 0 | sTM, P-sel, Fib,  vWf, | 6 |
| Marin F | 1999 | Spain | Case-control | 18 | 24 | AF  (disease control) | 56.0 | 51.0 | 22.2 | 12.5 | 0 | | | 0 | D-dimer, AT-III,  tPA, PAI | 6 |
|  |  |  |  | 18 | 20 | AF  (healthy control) | 56.0 | - | 22.2 | - | 0 | | | 0 |  |  |
| Li-Saw-Hee FL | 1999 | UK | Case-control | 25 | 25 | AF | - | - | - | - | - | | | - | sTM, P-sel, Fib,  vWf, D-dimer,  BTG | 6 |
| Minamino T | 1999 | Japan | Case-control | 28 | 28 | Chronic AF | 64.0 | 64.0 | 71.4 | 71.4 | 7.0 | | | 14.0 | BTG | 6 |
| Roldan V | 1998 | Spain | Case-control | 36 | 20 | Chronic AF | 62.0 | 62.0 | 44.4 | - | 0 | | | 0 | Fib, D-dimer,  AT-III, tPA, PAI | 7 |
| Tsai LM | 1998 | Taiwan | Case-control | 73 | 38 | Chronic AF | 65.0 | 63.0 | 75.3 | 73.7 | 11.0* | | | 0 | F1+2 | 6 |
| Minamino T | 1997 | Japan | Case-control | 45 | 45 | Chronic AF | 63.0 | 63.0 | 73.3 | 73.3 | - | | | - | Fib, D-dimer, tPA, PAI, BTG | 6 |
| Kahn SR | 1997 | Canada | Case-control | 50 | 31 | AF | - | - | - | - | 0 | | | 0 | PC, Fib, vWf | 7 |
| Sohara H | 1997 | Japan | Case-control | 21 | 9 | Paroxysmal AF | - | - | - | - | 0 | | | 0 | TAT, Fib, D-dimer, PF-4, BTG | 6 |
| Lip GY | 1996 | UK | Case-control | 51 | 26 | AF | - | - | - | - | 0 | | | 0 | PC, D-dimer | 6 |
| Lip GY | 1996 | UK | Case-control | 30 | 158 | Paroxysmal AF | - | - | - | - | 0 | | | 0 | Fib, D-dimer | 8 |
|  |  |  |  | 56 | 158 | Chronic AF | - | - | - | - | 0 | | | 0 | Fib, D-dimer |  |
| Mitusch R | 1996 | Germany | Case-control | 69 | 28 | AF | 71.0 | 70.0 | 42.0 | 60.7 | 0 | | | 0 | Fib, D-dimer,  AT-III, tPA, PAI,  F1+2 | 7 |
| Nagao T | 1995 | Japan | Case-control | 17 | 19 | AF | 81.5 | 78.4 | 47.1 | - | 0 | | | 0 | TAT, D-dimer,  PF-4, BTG | 8 |
| Lip GY | 1995 | UK | Case-control | 87 | 158 | Chronic AF | 63.0 | 59.3 | 50.6 | 55.7 | - | | | - | Fib, vWf, D-dimer, | 7 |
| Sohara H | 1994 | Japan | Case-control | 13 | 9 | Paroxysmal AF | 60.0 | - | 76.9 | - | 0 | | | 0 | TAT, Fib, D-dimer, PF-4, BTG | 6 |
| Kumagai K | 1990 | Japan | Case-control | 73 | 21 | Chronic AF | 64.0 | 61.0 | 53.4 | 42.9 | 0 | | | 0 | D-dimer | 7 |
| Gustafsson C | 1990 | Sweden | Case-control | 20 | 40 | AF with stroke | 77.0 | 77.0 | - | - | 0 | | | 0 | PC, Fib, vWf,  D-dimer, AT-III | 8 |
|  |  |  |  | 20 | 40 | AF without stroke | 77.0 | 77.0 | - | - | 0 | | | 0 | PC, Fib, vWf,  D-dimer, AT-III |  |
| Yamauchi K | 1986 | Japan | Case-control | 73 | 57 | AF without  valvular heart  disease | 55.0 | 36.0 | - | 89.5 | 0 | | | 0 | PF-4, BTG | 6 |
|  |  |  |  | 26 | 57 | AF with valvular  heart disease | 47.0 | 36.0 | - | 89.5 | 0 | | | 0 | PF-4, BTG |  |

NOS, Newcastle-Ottawa Scale; AF, atrial fibrillation; PC, platelet count; MPV, mean platelet volume; PF-4, platelet factor 4; BTG, β-thromboglobulin; P-sel, P-selectin; Fib, fibrinogen; TAT, thrombin–antithrombin; F1+2, prothombin fragments 1+2; AT- III, Antithrombin III; tPA, tissue plasminogen activator; PAI-1, plasminogen activator inhibitor-1; vWf, vonWillebrand factor; sTM, soluble thrombomodulin; -, not available.

**References**

1. Turgut O, Zorlu A, Kilicli F, Cinar Z, Yucel H, et al. (2013) Atrial fibrillation is associated with increased mean platelet volume in patients with type 2 diabetes mellitus. Platelets 24: 493-497.

2. Feng C, Mei W, Luo C, Long M, Hu X, et al. (2013) Relationship between mean platelet volume and coronary blood flow in patients with atrial fibrillation. Heart Lung Circ 22: 43-49.

3. Acevedo M, Corbalan R, Braun S, Pereira J, Gonzalez I, et al. (2012) Biochemical predictors of cardiac rhythm at 1 year follow-up in patients with non-valvular atrial fibrillation. J Thromb Thrombolysis 33:338-8.

4. Hayashi M, Takeshita K, Inden Y, Ishii H, Cheng XW, et al. (2011) Platelet activation and induction of tissue factor in acute and chronic atrial fibrillation: involvement of mononuclear cell-platelet interaction. Thromb Res 128: e113-118.

5. Zorlu A, Akkaya E, Altay H, Bektasoglu G, Turkdogan KA, et al. (2012) The relationship between D-dimer level and the development of atrial fibrillation in patients with systolic heart failure. J Thromb Thrombolysis 33: 343-348.

6. Adamsson Eryd S, Smith JG, Melander O, Hedblad B, Engstrom G (2011) Inflammation-sensitive proteins and risk of atrial fibrillation: a population-based cohort study. Eur J Epidemiol 26: 449-455.

7. Fu R, Wu S, Wu P, Qiu J (2011) A study of blood soluble P-selectin, fibrinogen, and von Willebrand factor levels in idiopathic and lone atrial fibrillation. Europace 13: 31-36.

8. Hou J, Liang Y, Gai X, Zhang H, Yang X, et al. (2010) The impact of acute atrial fibrillation on the prothrombotic state in patients with essential hypertension. Clin Biochem 43: 1212-1215.

9. Alonso A, Tang W, Agarwal SK, Soliman EZ, Chamberlain AM, et al. (2012) Hemostatic markers are associated with the risk and prognosis of atrial fibrillation: the ARIC study. Int J Cardiol 155: 217-222.

10. Schnabel RB, Larson MG, Yamamoto JF, Sullivan LM, Pencina MJ, et al. (2010) Relations of biomarkers of distinct pathophysiological pathways and atrial fibrillation incidence in the community. Circulation 121: 200-207.

11. Alberti S, Angeloni G, Tamburrelli C, Pampuch A, Izzi B, et al. (2009) Platelet-leukocyte mixed conjugates in patients with atrial fibrillation. Platelets 20: 235-241.

12. Gartner W, Zierhut B, Mineva I, Sodeck G, Leutmezer F, et al. (2008) Brain natriuretic peptide correlates with the extent of atrial fibrillation-associated silent brain lesions. Clin Biochem 41: 1434-1439.

13. Targonski R, Salczynska D, Sadowski J, Cichowski L (2008) Relationship between inflammatory markers and clinical patterns of atrial fibrillation in patients with congestive heart failure. Kardiol Pol 66: 729-736; discussion 737-729.

14. Choudhury A, Chung I, Panja N, Patel J, Lip GY (2008) Soluble CD40 ligand, platelet surface CD40 ligand, and total platelet CD40 ligand in atrial fibrillation: relationship to soluble P-selectin, stroke risk factors, and risk factor intervention. Chest 134: 574-581.

15. Colkesen Y, Acil T, Abayli B, Yigit F, Katircibasi T, et al. (2008) Mean platelet volume is elevated during paroxysmal atrial fibrillation: a marker of increased platelet activation? Blood Coagul Fibrinolysis 19: 411-414.

16. Marcus GM, Whooley MA, Glidden DV, Pawlikowska L, Zaroff JG, et al. (2008) Interleukin-6 and atrial fibrillation in patients with coronary artery disease: data from the Heart and Soul Study. Am Heart J 155: 303-309.

17. Blann AD, Choudhury A, Freestone B, Patel J, Lip GY (2008) Soluble CD40 ligand and atrial fibrillation: relationship to platelet activation, and endothelial damage/dysfunction. Int J Cardiol 127: 135-137.

18. Topaloglu S, Boyaci A, Ayaz S, Yilmaz S, Yanik O, et al. (2007) Coagulation, fibrinolytic system activation and endothelial dysfunction in patients with mitral stenosis and sinus rhythm. Angiology 58: 85-91.

19. Yip HK, Chang LT, Sun CK, Yang CH, Hung WC, et al. (2006) Platelet activation in patients with chronic nonvalvular atrial fibrillation. Int Heart J 47: 371-379.

20. Cecchi E, Marcucci R, Poli D, Antonucci E, Abbate R, et al. (2006) Hyperviscosity as a possible risk factor for cerebral ischemic complications in atrial fibrillation patients. Am J Cardiol 97: 1745-1748.

21. Turgut N, Akdemir O, Turgut B, Demir M, Ekuklu G, et al. (2006) Hypercoagulopathy in stroke patients with nonvalvular atrial fibrillation: hematologic and cardiologic investigations. Clin Appl Thromb Hemost 12: 15-20.

22. Heeringa J, Conway DS, van der Kuip DA, Hofman A, Breteler MM, et al. (2006) A longitudinal population-based study of prothrombotic factors in elderly subjects with atrial fibrillation: the Rotterdam Study 1990-1999. J Thromb Haemost 4: 1944-1949.

23. Roldan V, Marin F, Martinez JG, Garcia-Herola A, Sogorb F, et al. (2005) Relation of interleukin-6 levels and prothrombin fragment 1+2 to a point-based score for stroke risk in atrial fibrillation. Am J Cardiol 95: 881-882.

24. Marin F, Roldan V, Climent VE, Ibanez A, Garcia A, et al. (2004) Plasma von Willebrand factor, soluble thrombomodulin, and fibrin D-dimer concentrations in acute onset non-rheumatic atrial fibrillation. Heart 90: 1162-1166.

25. Inoue H, Nozawa T, Okumura K, Jong-Dae L, Shimizu A, et al. (2004) Prothrombotic activity is increased in patients with nonvalvular atrial fibrillation and risk factors for embolism. Chest 126: 687-692.

26. Conway DS, Buggins P, Hughes E, Lip GY (2004) Relationship of interleukin-6 and C-reactive protein to the prothrombotic state in chronic atrial fibrillation. J Am Coll Cardiol 43: 2075-2082.

27. Hatzinikolaou-Kotsakou E, Kartasis Z, Tziakas D, Hotidis A, Stakos D, et al. (2003) Atrial fibrillation and hypercoagulability: dependent on clinical factors or/and on genetic alterations? J Thromb Thrombolysis 16: 155-161.

28. Atalar E, Haznedaroglu IC, Acil T, Ozer N, Kilic H, et al. (2003) Patients with paroxysmal atrial fibrillation but not paroxysmal supraventricular tachycardia display evidence of platelet activation during arrhythmia. Platelets 14: 407-411.

29. Conway DS, Buggins P, Hughes E, Lip GY (2004) Relation of interleukin-6, C-reactive protein, and the prothrombotic state to transesophageal echocardiographic findings in atrial fibrillation. Am J Cardiol 93: 1368-1373, A1366.

30. Kamath S, Blann AD, Chin BS, Lip GY (2003) Platelet activation, haemorheology and thrombogenesis in acute atrial fibrillation: a comparison with permanent atrial fibrillation. Heart 89: 1093-1095.

31. Marin F, Roldan V, Climent V, Garcia A, Marco P, et al. (2003) Is thrombogenesis in atrial fibrillation related to matrix metalloproteinase-1 and its inhibitor, TIMP-1? Stroke 34: 1181-1186.

32. Conway DS, Heeringa J, Van Der Kuip DA, Chin BS, Hofman A, et al. (2003) Atrial fibrillation and the prothrombotic state in the elderly: the Rotterdam Study. Stroke 34: 413-417.

33. Kamath S, Chin BS, Blann AD, Lip GY (2002) A study of platelet activation in paroxysmal, persistent and permanent atrial fibrillation. Blood Coagul Fibrinolysis 13: 627-636.

34. Kamath S, Blann AD, Chin BS, Lanza F, Aleil B, et al. (2002) A study of platelet activation in atrial fibrillation and the effects of antithrombotic therapy. Eur Heart J 23: 1788-1795.

35. Kamath S, Blann AD, Caine GJ, Gurney D, Chin BS, et al. (2002) Platelet P-selectin levels in relation to plasma soluble P-selectin and beta-thromboglobulin levels in atrial fibrillation. Stroke 33: 1237-1242.

36. Wang TD, Chen WJ, Su SS, Su TC, Chen MF, et al. (2001) Increased levels of tissue plasminogen activator antigen and factor VIII activity in nonvalvular atrial fibrillation: relation to predictors of thromboembolism. J Cardiovasc Electrophysiol 12: 877-884.

37. Li-Saw-Hee FL, Blann AD, Gurney D, Lip GY (2001) Plasma von Willebrand factor, fibrinogen and soluble P-selectin levels in paroxysmal, persistent and permanent atrial fibrillation. Effects of cardioversion and return of left atrial function. Eur Heart J 22: 1741-1747.

38. Feng D, D'Agostino RB, Silbershatz H, Lipinska I, Massaro J, et al. (2001) Hemostatic state and atrial fibrillation (the Framingham Offspring Study). Am J Cardiol 87: 168-171.

39. Topcuoglu MA, Haydari D, Ozturk S, Ozcebe OI, Saribas O (2000) Plasma levels of coagulation and fibrinolysis markers in acute ischemic stroke patients with lone atrial fibrillation. Neurol Sci 21: 235-240.

40. Mondillo S, Sabatini L, Agricola E, Ammaturo T, Guerrini F, et al. (2000) Correlation between left atrial size, prothrombotic state and markers of endothelial dysfunction in patients with lone chronic nonrheumatic atrial fibrillation. Int J Cardiol 75: 227-232.

41. Giansante C, Fiotti N, Miccio M, Altamura N, Salvi R, et al. (2000) Coagulation indicators in patients with paroxysmal atrial fibrillation: effects of electric and pharmacologic cardioversion. Am Heart J 140: 423-429.

42. Li-Saw-Hee FL, Blann AD, Lip GY (2000) A cross-sectional and diurnal study of thrombogenesis among patients with chronic atrial fibrillation. J Am Coll Cardiol 35: 1926-1931.

43. Marin F, Roldan V, Monmeneu JV, Bodi V, Fernandez C, et al. (1999) Prothrombotic state and elevated levels of plasminogen activator inhibitor-1 in mitral stenosis with and without atrial fibrillation. Am J Cardiol 84: 862-864, A869.

44. Li-Saw-Hee FL, Blann AD, Goldsmith I, Lip GY (1999) Indexes of hypercoagulability measured in peripheral blood reflect levels in intracardiac blood in patients with atrial fibrillation secondary to mitral stenosis. Am J Cardiol 83: 1206-1209.

45. Minamino T, Kitakaze M, Asanuma H, Ueda Y, Koretsune Y, et al. (1999) Plasma adenosine levels and platelet activation in patients with atrial fibrillation. Am J Cardiol 83: 194-198.

46. Roldan V, Marin F, Marco P, Martinez JG, Calatayud R, et al. (1998) Hypofibrinolysis in atrial fibrillation. Am Heart J 136: 956-960.

47. Tsai LM, Chen JH, Tsao CJ (1998) Relation of left atrial spontaneous echo contrast with prethrombotic state in atrial fibrillation associated with systemic hypertension, idiopathic dilated cardiomyopathy, or no identifiable cause (lone). Am J Cardiol 81: 1249-1252.

48. Minamino T, Kitakaze M, Sato H, Asanuma H, Funaya H, et al. (1997) Plasma levels of nitrite/nitrate and platelet cGMP levels are decreased in patients with atrial fibrillation. Arterioscler Thromb Vasc Biol 17: 3191-3195.

49. Kahn SR, Solymoss S, Flegel KM (1997) Nonvalvular atrial fibrillation: evidence for a prothrombotic state. CMAJ 157: 673-681.

50. Sohara H, Amitani S, Kurose M, Miyahara K (1997) Atrial fibrillation activates platelets and coagulation in a time-dependent manner: a study in patients with paroxysmal atrial fibrillation. J Am Coll Cardiol 29: 106-112.

51. Lip GY, Lip PL, Zarifis J, Watson RD, Bareford D, et al. (1996) Fibrin D-dimer and beta-thromboglobulin as markers of thrombogenesis and platelet activation in atrial fibrillation. Effects of introducing ultra-low-dose warfarin and aspirin. Circulation 94: 425-431.

52. Lip GY, Lowe GD, Rumley A, Dunn FG (1996) Fibrinogen and fibrin D-dimer levels in paroxysmal atrial fibrillation: evidence for intermediate elevated levels of intravascular thrombogenesis. Am Heart J 131: 724-730.

53. Mitusch R, Siemens HJ, Garbe M, Wagner T, Sheikhzadeh A, et al. (1996) Detection of a hypercoagulable state in nonvalvular atrial fibrillation and the effect of anticoagulant therapy. Thromb Haemost 75: 219-223.

54. Nagao T, Hamamoto M, Kanda A, Tsuganesawa T, Ueda M, et al. (1995) Platelet activation is not involved in acceleration of the coagulation system in acute cardioembolic stroke with nonvalvular atrial fibrillation. Stroke 26: 1365-1368.

55. Lip GY, Lowe GD, Rumley A, Dunn FG (1995) Increased markers of thrombogenesis in chronic atrial fibrillation: effects of warfarin treatment. Br Heart J 73: 527-533.

56. Sohara H, Miyahara K (1994) Effect of atrial fibrillation on the fibrino-coagulation system--study in patients with paroxysmal atrial fibrillation. Jpn Circ J 58: 821-826.

57. Kumagai K, Fukunami M, Ohmori M, Kitabatake A, Kamada T, et al. (1990) Increased intracardiovascular clotting in patients with chronic atrial fibrillation. J Am Coll Cardiol 16: 377-380.

58. Gustafsson C, Blomback M, Britton M, Hamsten A, Svensson J (1990) Coagulation factors and the increased risk of stroke in nonvalvular atrial fibrillation. Stroke 21: 47-51.

59. Yamauchi K, Furui H, Taniguchi N, Sotobata I (1986) Plasma beta-thromboglobulin and platelet factor 4 concentrations in patients with atrial fibrillation. Jpn Heart J 27: 481-487.
