# Supplementary material for: Association of Hemostatic Markers with Atrial Fibrillation: A Meta-Analysis and Meta-Regression
Source: PLoS One. 2015 Apr 17;10(4):e0124716. doi: 10.1371/journal.pone.0124716 (PMC4401562; doi:10.1371/journal.pone.0124716)
Supplement: S3 Table — (DOC) [file pone.0124716.s013.doc]

**Table S3. Univariate meta-regression results for haemostatic markers**

| **Confounding factors** | **Regression coefficient** | **Standard error** | **95% CI**  **of coefficient** | **P**  **value** | **Tau^2^** | **Adj R^2^**  **(%)** |
| --- | --- | --- | --- | --- | --- | --- |
| **Platelet count** |  |  |  |  |  |  |
| Study design | － | － | － | － | － | － |
| Publication year | 0.88 | 0.83 | -0.87, 2.62 | 0.30 | 1.80 | 2.81 |
| Mean age | -0.96 | 1.11 | -3.31, 1.39 | 0.40 | 1.90 | -2.10 |
| Gender | 0.63 | 1.05 | -1.66, 2.92 | 0.56 | 2.12 | -8.46 |
| Type of AF |  |  |  |  |  |  |
| Paroxysmal AF | 6.24 | 0.73 | 3.93, 8.56 | 0.01 | 0.01 | 99.78 |
| Persistent AF | － | － | － | － | － | － |
| Permanent AF | 6.15 | 0.73 | 3.81, 8.48 | 0.01 | 0.01 | 99.78 |
| Hypertension | -0.91 | 0.29 | -1.59, -0.23 | 0.02 | 0.47 | 56.20 |
| Coronary artery disease | -0.72 | 0.24 | -1.32, -0.13 | 0.02 | 0.49 | 59.52 |
| Cerebrovascular accidents | -0.87 | 1.17 | -2.40, 4.13 | 0.50 | 0.77 | -14.04 |
| Diabetes mellitus | -0.90 | 0.44 | -1.93, 0.12 | 0.08 | 0.76 | 28.65 |
| Smoking | -1.17 | 0.60 | -2.83, 0.49 | 0.12 | 1.03 | 36.78 |
|  |  |  |  |  |  |  |
| **MPV** |  |  |  |  |  |  |
| Study design | － | － | － | － | － | － |
| Publication year | － | － | － | － | － | － |
| Mean age | -0.02 | 1.59 | -5.07, 5.03 | 0.99 | 1.98 | -34.76 |
| Gender | -0.12 | 1.05 | -3.47, 3.24 | 0.92 | 1.97 | -34.26 |
| Type of AF | － | － | － | － | － | － |
| Hypertension | 0.60 | 1.56 | -4.37, 5.56 | 0.73 | 1.88 | -28.38 |
| Coronary artery disease | 0.06 | 1.90 | -8.10, 8.22 | 0.98 | 2.98 | -51.50 |
| Cerebrovascular accidents | 0.05 | 2.70 | -11.55, 11.64 | 0.99 | 2.98 | -51.55 |
| Diabetes mellitus | 0.12 | 1.22 | -3.75, 3.99 | 0.93 | 1.97 | -34.35 |
| Smoking | － | － | － | － | － | － |
|  |  |  |  |  |  |  |
| **PF-4** |  |  |  |  |  |  |
| Study design | － | － | － | － | － | － |
| Publication year | 3.28 | 2.21 | -1.54, 8.09 | 0.16 | 12.61 | 13.63 |
| Mean age | -1.97 | 2.43 | -7.29, 3.34 | 0.43 | 15.98 | -9.44 |
| Gender | 3.17 | 10.06 | -124.62, 130.97 | 0.81 | 0.20 | -338.71 |
| Type of AF | － | － | － | － | － | － |
| Hypertension | -0.44 | 0.22 | -1.38, 0.50 | 0.18 | 0.03 | 83.61 |
| Coronary artery disease | － | － | － | － | － | － |
| Cerebrovascular accidents | － | － | － | － | － | － |
| Diabetes mellitus | － | － | － | － | － | － |
| Smoking | -1.39 | 0.75 | -10.86, 8.07 | 0.31 | 0.14 | 66.35 |
|  |  |  |  |  |  |  |
| **BTG** |  |  |  |  |  |  |
| Study design | － | － | － | － | － | － |
| Publication year | 1.50 | 0.84 | -0.26, 3.26 | 0.09 | 3.74 | 9.51 |
| Mean age | 0.32 | 1.18 | -2.13, 2.77 | 0.79 | 4.34 | -4.95 |
| Gender | -0.66 | 0.65 | -2.15, 0.83 | 0.34 | 0.15 | -7.18 |
| Type of AF |  |  |  |  |  |  |
| Paroxysmal AF | -0.29 | 0.41 | -1.42, 0.84 | 0.52 | 0.12 | -61.59 |
| Persistent AF | － | － | － | － | － | － |
| Permanent AF | － | － | － | － | － | － |
| Hypertension | 0.03 | 0.21 | -0.50, 0.55 | 0.91 | 0.30 | -23.06 |
| Coronary artery disease | 0.07 | 0.16 | -0.38, 0.52 | 0.68 | 0.22 | -29.30 |
| Cerebrovascular accidents | － | － | － | － | － | － |
| Diabetes mellitus | -0.09 | 0.23 | -0.72, 0.55 | 0.73 | 0.22 | -26.17 |
| Smoking | 0.31 | 0.29 | -0.45, 1.06 | 0.34 | 0.16 | -2.32 |
|  |  |  |  |  |  |  |
| **P-selectin** |  |  |  |  |  |  |
| Study design | -0.63 | 0.69 | -2.06, 0.80 | 0.37 | 0.73 | -0.78 |
| Publication year | 1.78 | 0.92 | -0.15, 3.71 | 0.07 | 0.60 | 17.62 |
| Mean age | -0.08 | 0.98 | -2.12, 1.96 | 0.93 | 0.78 | -7.29 |
| Gender | -1.01 | 1.02 | -3.18, 1.15 | 0.34 | 0.70 | -2.97 |
| Type of AF |  |  |  |  |  |  |
| Paroxysmal AF | -0.80 | 1.39 | -4.37, 2.78 | 0.59 | 1.71 | -30.83 |
| Persistent AF | － | － | － | － | － | － |
| Permanent AF | 0.25 | 0.99 | -4.37, 2.78 | 0.59 | 1.71 | -30.83 |
| Hypertension | -0.85 | 0.51 | -1.94, 0.25 | 0.12 | 0.79 | 16.55 |
| Coronary artery disease | -0.90 | 0.84 | -2.8, 1.00 | 0.31 | 0.97 | 2.69 |
| Cerebrovascular accidents | -1.54 | 1.67 | -5.84, 2.77 | 0.40 | 1.57 | -3.58 |
| Diabetes mellitus | -0.79 | 0.59 | -2.11, 0.53 | 0.21 | 0.83 | 10.77 |
| Smoking | 0.58 | 0.54 | -0.61, 1.77 | 0.31 | 1.02 | 3.12 |
|  |  |  |  |  |  |  |
| **D-dimer** |  |  |  |  |  |  |
| Study design | － | － | － | － | － | － |
| Publication year | 0.51 | 0.75 | -1.02, 2.03 | 0.50 | 4.66 | -1.87 |
| Mean age | 0.63 | 1.18 | -1.77, 3.03 | 0.59 | 4.68 | -2.26 |
| Gender | -2.52 | 1.31 | -5.27, 0.23 | 0.07 | 2.34 | 13.18 |
| Type of AF |  |  |  |  |  |  |
| Paroxysmal AF | -0.38 | 0.30 | -1.15, 0.38 | 0.25 | 0.07 | 92.36 |
| Persistent AF | 2.66 | 0.58 | 1.17, 4.15 | 0.01 | 0.07 | 92.36 |
| Permanent AF | － | － | － | － | － | － |
| Hypertension | 0.03 | 0.49 | -1.04, 1.09 | 0.96 | 2.49 | -9.10 |
| Coronary artery disease | -0.14 | 0.60 | -1.67, 1.40 | 0.83 | 4.14 | -20.57 |
| Cerebrovascular accidents | － | － | － | － | － | － |
| Diabetes mellitus | -0.17 | 0.70 | -1.79, 1.44 | 0.81 | 3.76 | -12.39 |
| Smoking | 0.19 | 0.20 | -0.27, 0.66 | 0.36 | 0.10 | -7.04 |
|  |  |  |  |  |  |  |
| **Fibrinogen** |  |  |  |  |  |  |
| Study design | -0.60 | 0.42 | -1.44, 0.24 | 0.16 | 0.93 | 2.92 |
| Publication year | -1.11 | 0.32 | -1.74, -0.47 | 0.01 | 0.69 | 27.57 |
| Mean age | 0.37 | 0.39 | -0.42, 1.15 | 0.35 | 0.96 | -0.51 |
| Gender | -1.81 | 0.73 | -3.29, -0.33 | 0.02 | 0.96 | 15.39 |
| Type of AF |  |  |  |  |  |  |
| Paroxysmal AF | -0.25 | 0.79 | -1.98, 1.48 | 0.75 | 0.71 | -13.62 |
| Persistent AF | － | － | － | － | － | － |
| Permanent AF | -0.53 | 0.79 | -1.98, 1.48 | 0.75 | 0.71 | -13.62 |
| Hypertension | -0.07 | 0.34 | -0.77, 0.62 | 0.83 | 1.37 | -4.58 |
| Coronary artery disease | 0.08 | 0.34 | -0.62, 0.78 | 0.82 | 1.81 | -5.87 |
| Cerebrovascular accidents | -1.80 | 1.54 | -5.29, 1.69 | 0.27 | 0.76 | 4.15 |
| Diabetes mellitus | -0.12 | 0.27 | -0.68, 0.44 | 0.66 | 1.34 | -3.77 |
| Smoking | 0.34 | 0.31 | -0.29, 0.98 | 0.28 | 0.53 | 1.08 |
|  |  |  |  |  |  |  |
| **TAT** |  |  |  |  |  |  |
| Study design | － | － | － | － | － | － |
| Publication year | -2.78 | 0.35 | -1.40, 0.85 | 0.49 | 0.04 | 5.68 |
| Mean age | － | － | － | － | － | － |
| Gender | － | － | － | － | － | － |
| Type of AF | － | － | － | － | － | － |
| Hypertension | － | － | － | － | － | － |
| Coronary artery disease | － | － | － | － | － | － |
| Cerebrovascular accidents | － | － | － | － | － | － |
| Diabetes mellitus | － | － | － | － | － | － |
| Smoking | － | － | － | － | － | － |
|  |  |  |  |  |  |  |
| **F1+2** |  |  |  |  |  |  |
| Study design | － | － | － | － | － | － |
| Publication year | -2.59 | 0.85 | -4.61, -0.57 | 0.02 | 1.05 | 51.97 |
| Mean age | － | － | － | － | － | － |
| Gender | 0.60 | 1.81 | -4.41, 5.62 | 0.76 | 1.87 | -23.58 |
| Type of AF | － | － | － | － | － | － |
| Hypertension | 0.05 | 0.87 | -2.72, 2.83 | 0.96 | 0.30 | -41.50 |
| Coronary artery disease | － | － | － | － | － | － |
| Cerebrovascular accidents | － | － | － | － | － | － |
| Diabetes mellitus | -0.21 | 0.24 | -0.96, 0.54 | 0.43 | 0.22 | -5.19 |
| Smoking | － | － | － | － | － | － |
|  |  |  |  |  |  |  |
| **AT- III** |  |  |  |  |  |  |
| Study design | － | － | － | － | － | － |
| Publication year | － | － | － | － | － | － |
| Mean age | -0.26 | 2.03 | -5.90, 5.38 | 0.91 | 3.29 | -25.62 |
| Gender | － | － | － | － | － | － |
| Type of AF | － | － | － | － | － | － |
| Hypertension | -2.05 | 1.46 | -20.57, 16.48 | 0.39 | 4.37 | 33.07 |
| Coronary artery disease | -0.90 | 1.18 | -15.89, 14.08 | 0.58 | 8.30 | -27.13 |
| Cerebrovascular accidents | － | － | － | － | － | － |
| Diabetes mellitus | -3.93 | 3.30 | -45.86, 38.01 | 0.45 | 5.40 | 17.18 |
| Smoking | － | － | － | － | － | － |
|  |  |  |  |  |  |  |
| **tPA** |  |  |  |  |  |  |
| Study design | 1.68 | 1.66 | -2.08, 5.44 | 0.34 | 2.43 | 0.69 |
| Publication year | 0.43 | 1.02 | -1.87, 2.73 | 0.68 | 2.68 | -9.27 |
| Mean age | -0.04 | 1.06 | -2.44, 2.36 | 0.97 | 2.74 | -11.87 |
| Gender | -3.59 | 1.81 | -8.25, 1.06 | 0.10 | 2.11 | 33.58 |
| Type of AF | － | － | － | － | － | － |
| Hypertension | -1.49 | 1.90 | -7.55, 4.57 | 0.49 | 4.32 | -11.20 |
| Coronary artery disease | -1.05 | 1.36 | -6.91, 4.81 | 0.52 | 5.24 | -16.52 |
| Cerebrovascular accidents | － | － | － | － | － | － |
| Diabetes mellitus | -1.04 | 1.28 | -5.13, 3.04 | 0.48 | 4.27 | -9.76 |
| Smoking | -2.84 | 2.28 | -10.10, 4.42 | 0.30 | 0.86 | 13.28 |
|  |  |  |  |  |  |  |
| **PAI-1** |  |  |  |  |  |  |
| Study design | -0.45 | 1.18 | -3.02, 2.12 | 0.71 | 2.35 | -7.60 |
| Publication year | 0.11 | 0.84 | -1.72, 1.94 | 0.90 | 2.37 | -8.53 |
| Mean age | 1.04 | 0.82 | -0.75, 2.83 | 0.23 | 2.09 | 4.39 |
| Gender | -1.89 | 1.81 | -6.32, 2.54 | 0.34 | 2.29 | 1.35 |
| Type of AF | － | － | － | － | － | － |
| Hypertension | -0.84 | 0.73 | -2.62, 0.95 | 0.30 | 2.99 | 4.19 |
| Coronary artery disease | -0.84 | 0.63 | -2.59, 0.92 | 0.26 | 3.73 | 13.32 |
| Cerebrovascular accidents | － | － | － | － | － | － |
| Diabetes mellitus | -0.99 | 1.10 | -3.81, 1.84 | 0.41 | 3.79 | -3.70 |
| Smoking | -1.49 | 2.28 | -7.34, 4.36 | 0.54 | 3.64 | -10.90 |
|  |  |  |  |  |  |  |
| **vWf** |  |  |  |  |  |  |
| Study design | -0.69 | 0.32 | -1.35, -0.03 | 0.04 | 0.32 | 16.10 |
| Publication year | -0.49 | 0.33 | -1.16, 0.19 | 0.15 | 0.35 | 8.45 |
| Mean age | 0.36 | 0.31 | -0.28, 0.99 | 0.26 | 0.37 | 1.87 |
| Gender | -2.59 | 1.07 | -4.81, -0.36 | 0.03 | 0.35 | 23.00 |
| Type of AF |  |  |  |  |  |  |
| Paroxysmal AF | 0.26 | 1.00 | -2.55, 3.06 | 0.81 | 0.87 | -51.79 |
| Persistent AF | － | － | － | － | － | － |
| Permanent AF | 0.64 | 0.92 | -2.55, 3.06 | 0.25 | 0.87 | -51.79 |
| Hypertension | -0.23 | 0.18 | -0.61, 0.15 | 0.22 | 0.25 | 5.38 |
| Coronary artery disease | -0.04 | 0.20 | -0.49, 0.40 | 0.84 | 0.39 | -14.18 |
| Cerebrovascular accidents | -1.97 | 1.93 | -6.70, 2.76 | 0.35 | 0.60 | 0.56 |
| Diabetes mellitus | -0.31 | 0.28 | -0.90, 1.32 | 0.27 | 0.32 | 3.00 |
| Smoking | 0.49 | 0.33 | -0.19, 1.18 | 0.15 | 0.37 | 10.91 |
|  |  |  |  |  |  |  |
| **sTM** |  |  |  |  |  |  |
| Study design |  |  |  |  |  |  |
| Publication year | 2.11 | 0.78 | -0.06, 4.28 | 0.05 | 0.41 | 61.10 |
| Mean age | － | － | － | － | － | － |
| Gender | -3.96 | 4.75 | -24.42, 16.50 | 0.49 | 0.30 | -13.51 |
| Type of AF | － | － | － | － | － | － |
| Hypertension | － | － | － | － | － | － |
| Coronary artery disease | － | － | － | － | － | － |
| Cerebrovascular accidents | － | － | － | － | － | － |
| Diabetes mellitus | － | － | － | － | － | － |
| Smoking | － | － | － | － | － | － |

Univariate random effect meta-regression analysis was performed. Tau^2^ is the REML estimate of between-study variance. R^2^ is the proportion of between-study variance explained by the covariates. “－”, Some indexes are not available because meta-regression analysis was not performed due to limited number of studies. MPV, mean platelet volume; PF-4, platelet factor-4; BTG, β-thromboglobulin; TAT, thrombin-antithrombin; F1+2, prothrombin fragment 1+2; AT- III, antithrombin-III; tPA, tissue-type plasminogen activator; PAI-1, plasminogen activator inhibitor-1; vWf, vonWillebrand factor; sTM, soluble thrombomodulin; CI, confidence interval; Adj, adjusted.
