# Supplementary material for: Association of Hemostatic Markers with Atrial Fibrillation: A Meta-Analysis and Meta-Regression
Source: PLoS One. 2015 Apr 17;10(4):e0124716. doi: 10.1371/journal.pone.0124716 (PMC4401562; doi:10.1371/journal.pone.0124716)
Supplement: S4 Table — (DOC) [file pone.0124716.s014.doc]

**Table S4. Multivariate meta-regression results for fibrinogen and vWf**

| Confounding factors | Regression coefficient | Standard error | 95% CI  of coefficient | P  value | Tau^2^ | Adj R^2^  (%) | P for test for model |
| --- | --- | --- | --- | --- | --- | --- | --- |
| **Fibrinogen** |  |  |  |  |  |  |  |
| Publication year | -1.98 | 0.57 | -3.14, -0.82 | 0.001 | 0.67 | 41.26 | 0.0004 |
| Gender | -1.29 | 0.63 | -2.58, 0.01 | 0.05 |
|  |  |  |  |  |  |  |  |
| **vWf** |  |  |  |  |  |  |  |
| Study design | -0.41 | 0.39 | -1.22, 0.40 | 0.30 | 0.34 | 23.89 | 0.0505 |
| Gender | -2.00 | 1.20 | -4.51, 0.53 | 0.11 |

Multivariate random effect meta-regression analysis was performed. Tau^2^ is the REML estimate of between-study variance. Adj R^2^ is the proportion of between-study variance explained by the covariates. vWf, vonWillebrand factor; CI, confidence interval; Adj, adjusted.
